# Analysis of drought-responsive signalling network in two contrasting rice cultivars using transcriptome-based approach

**DOI:** 10.1038/srep42131

**Published:** 2017-02-09

**Authors:** Pratikshya Borah, Eshan Sharma, Amarjot Kaur, Girish Chandel, Trilochan Mohapatra, Sanjay Kapoor, Jitendra P. Khurana

**Affiliations:** 1 Interdisciplinary Centre for Plant Genomics, University of Delhi South Campus, New Delhi - 110021, India; 2 Department of Plant Molecular Biology, University of Delhi South Campus, New Delhi - 110021, India; 3 Indira Gandhi Agricultural University, Raipur **-** 492012, India; 4 Department of Agricultural Research and Education, Indian Council of Agricultural Research, New Delhi - 110012, India

## Abstract

Traditional cultivars of rice in India exhibit tolerance to drought stress due to their inherent genetic variations. Here we present comparative physiological and transcriptome analyses of two contrasting cultivars, drought tolerant Dhagaddeshi (DD) and susceptible IR20. Microarray analysis revealed several differentially expressed genes (DEGs) exclusively in DD as compared to IR20 seedlings exposed to 3 h drought stress. Physiologically, DD seedlings showed higher cell membrane stability and differential ABA accumulation in response to dehydration, coupled with rapid changes in gene expression. Detailed analyses of metabolic pathways enriched in expression data suggest interplay of ABA dependent along with secondary and redox metabolic networks that activate osmotic and detoxification signalling in DD. By co-localization of DEGs with QTLs from databases or published literature for physiological traits of DD and IR20, candidate genes were identified including those underlying major QTL *qDTY*_*1.1*_ in DD. Further, we identified previously uncharacterized genes from both DD and IR20 under drought conditions including *OsWRKY51, OsVP1* and confirmed their expression by qPCR in multiple rice cultivars. *OsFBK1* was also functionally validated in susceptible PB1 rice cultivar and *Arabidopsis* for providing drought tolerance. Some of the DEGs mapped to the known QTLs could thus, be of potential significance for marker-assisted breeding.

Rice (*Oryza sativa* L.) is considered a staple food crop and is consumed by more than half of the world’s population. The Green Revolution movement in various countries heralded the accelerated production of this cereal crop. However, like in case of other crops, both abiotic and biotic factors affect the growth and development of rice, adversely affecting its productivity. Further, stagnating yield of rice cultivars along with climate change-related hazards are causing major concern for world food security. Historically, rice cultivars have been grown in areas irrigated essentially by floods. This makes rice more sensitive to changes in soil water content as compared to other cereals like maize and wheat as rice requires copious amount of water for its production. Consequently, drought is the most severe stress for rice production in rain-fed areas of more than 20 million hectare in South and Southeast Asia^1^ thereby adversely affecting popular high-yielding, albeit drought sensitive rice cultivars like Swarna, IR64 and MTU1010 grown in these areas^2^,^3^. With mounting pressure on food grain production, improvement in water use efficiency of rice cultivars is gaining worldwide attention, and the focus has shifted to the identification of cultivars that demonstrate increased yield under drought stress conditions. In recent years, bio-prospecting of rice cultivars better adapted to various abiotic stresses has been initiated in several countries^4^,^5^,^6^,^7^,^8^.

Rice cultivars found traditionally in India have many desirable characteristics and some of them do indeed exhibit differential responses to abiotic and biotic stresses. Indigenous cultivars like Dhagaddeshi and Nagina22 have been found to be drought tolerant, although low-yielding as opposed to the commercial cultivars. A preferred breeding strategy to improve drought tolerance involves the identification and introgression of QTLs for grain yield under drought conditions^9^. For example, by crossing Dhagaddeshi with Swarna and IR64 (drought susceptible and high yielding), a major-effect QTL, *qDTY1.1*, was identified on chromosome 1 that is characteristically associated with regulating grain yield under drought stress conditions^9^. Apart from *qDTY1.1*, several other QTLs have also been investigated for their potential to confer drought tolerance^10^,^11^. IR20 is an *indica* cultivar with short stature, shallow root system and high yield potential that makes it an elite genotype for crop production. However, it is susceptible to moisture stress and hence, there is a growing concern for its yield under prolonged dehydration. This is true for many other cultivars of rice and there is thus need to unravel the molecular mechanism(s) that are essentially responsible for making a cultivar either tolerant or susceptible to drought or for that matter to various abiotic stresses, since some of the underlying mechanisms are likely to be common.

Transcriptome analysis of rice in response to various abiotic stresses has been carried out in the past that led to the identification of a large number of stress-responsive genes^12^,^13^,^14^,^15^,^16^,^17^,^18^. Such studies have identified a large number of transcription factors, genes encoding for osmolyte production, reactive oxygen species (ROS) scavenging and other metabolic pathways etc. that could facilitate the selection of candidate genes for developing crop plants better adapted to abiotic stress conditions^19^. These genes can be broadly divided into two groups, viz. signalling component and functional component^20^. Efforts have been made to further characterize such stress-responsive genes to decipher the abiotic stress regulatory networks in rice. Although various approaches have been adopted to build up the current repository of information, only few studies have attempted to study the underlying pathways operative under stress. Therefore, it is imperative to decipher the intricacies of the regulatory networks associated with abiotic stress response in rice by adopting a more holistic approach.

The present study deals with the microarray based transcriptome analysis of Dhagaddeshi (drought tolerant) and IR20 (drought sensitive) seedlings subjected to drought stress conditions for different durations. Although newer technologies are now available for transcriptome analyses, microarray still accounts for being a robust and reliable method for such studies, particularly for rice, because the finished-quality genome sequence available for rice was used to develop these microarray chips. Our group in fact participated in sequencing the rice genome (IRGSP 2005) and also used these first generation rice microarray chips extensively for identifying genes involved in regulating reproductive development, hormone signalling and abiotic stress response^21^,^22^,^23^,^24^. We have attempted to dissect the signalling networks operating in these contrasting drought responsive cultivars by employing several down-stream analyses to obtain a holistic picture, with the aim to eventually identify genes unique to both cultivars, functionally validate them in transgenics, and also exploit them in molecular breeding strategies. We have also functionally validated a previously uncharacterised gene, *OsFBK1*, in *Arabidopsis* (mutant and over-expression) and in Pusa Basmati 1 (PB1) cultivar of rice by raising both knock-down and over-expression transgenics; to provide proof of concept of our present study and how it could be used to mine potential genes from both cultivars to explore their capabilities in providing drought tolerance.

## Results

### Comparative physiological and differential gene expression analyses of DD and IR20 under stress

**Figure 1:**
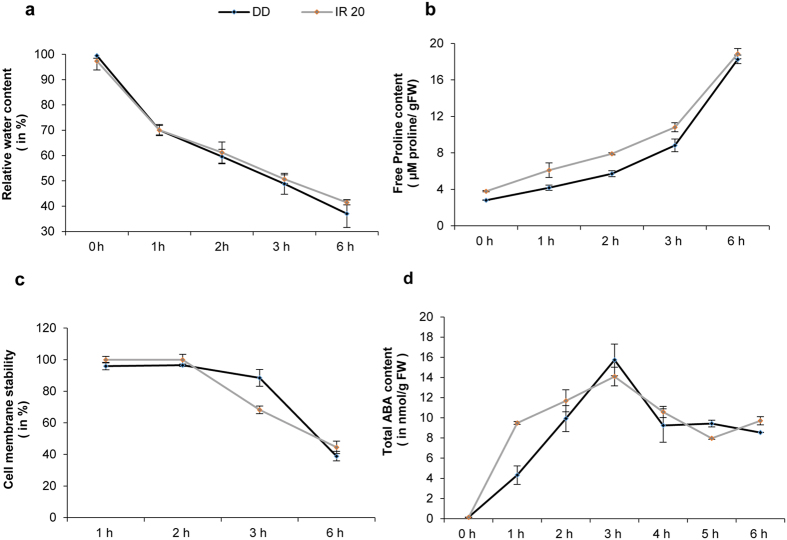
Comparative physiological analysis of Dhagaddeshi and IR20. (**a**) Relative water content, (**b**) Free Proline content. (**c**) Cell membrane stability. (**d**) Total estimated ABA content of Dhagaddeshi and IR20 seedlings under drought stress at specified time points in hours. All the experiments were done in triplicates and the mean values (±SE) were plotted against duration of drought stress treatment in hours.

For physiological analysis of Dhagaddeshi (DD) and IR20, seedlings were grown hydroponically for 7 days and given water deficit stress as described previously^13^. Seedlings of both cultivars showed similar decrease in relative water content (RWC) during stress treatment with highest change observed just after 1 hour of stress (Fig. 1a). Change in RWC close to 50% level was observed after 3 h of stress treatment in both cultivars (48.9% in DD and 50.7% in IR20). Drought stress is known to induce accumulation of osmolytes such as proline, glycine betaine that help in the prevention of dehydration in plants. A significant increase in the accumulation of free proline was observed in both cultivars as the stress duration progressed (Fig. 1b). Seedlings of DD exposed to drought stress showed higher percentage of cell membrane stability or, in other words, lower ion leakage after 3 h of stress. ABA is known to accumulate under stress and trigger the stress responses in plants and thus was also quantified. DD and IR20 seedlings showed a differential ABA accumulation pattern under water deficit stress; the seedlings of IR20 had more content of ABA than DD at 3 h post desiccation stress even though no further increase in ABA content was observed in both the cultivars on prolonged stress (Fig. 1d). The changes in total chlorophyll and carotenoid content in seedlings under stress appeared to be similar in both DD and IR20 seedlings (Supplementary Fig. S1).

### Differential gene expression analysis of DD and IR20 seedlings under drought stress

The 7-day-old seedlings of both the cultivars were subjected to drought stress as described earlier and microarray hybridization of the RNA isolated from samples collected after 3 h and 6 h along with that of control seedlings, was carried out as per manufacturer’s instructions (see Methods for more details). Following washing and scanning, the robustness of the microarray data was first checked by performing Principal Component Analysis (PCA). The replicates of each sample were found to be grouped together and all the replicates of DD and IR20 formed clusters largely distinct from each other (Supplementary Fig. S2). The diffex analysis (p < 0.05, >2-fold change) of the normalized and log transformed data revealed the number of probe sets expressing differentially after 3 h stress is almost double for DD (10,901) w.r.t. its control as compared to the same for IR20 (5,502) (Supplementary Fig. S3A). The differences in probe set numbers corresponding to differentially expressing genes after 6 h of stress was 8,601 in case of IR20 vis-à-vis 11,041 in DD. It could be assumed that the changes brought about in the transcriptome in DD are more significant in terms of the activation of the initial responses to stress within 3 h of exposure to desiccation than IR20. Despite the initial delay in sensing stress by IR20, the differences in the transcript levels under drought conditions in both the cultivars were more or less mitigated at the 6 h time point.

Therefore, based on comparative physiological analysis between both cultivars, and the fact that RWC for both cultivars at 3 h stress was essentially similar, and due to the above-mentioned reasons, the 3 h time-frame was chosen for detailed gene expression analysis. The 3 h time point would also enable the detection of genes involved in the early signalling responses to reduction of 50% water content on exposure to drought stress.

From the list of probe sets obtained after microarray data analysis at the 3 h stress time point in both the cultivars; the number of genes were manually sourced (Methods). The number of genes obtained after manual curation is shown in Supplementary Fig. S3B. While the genes highlighted for each cultivar at 3 h stress were compared with the respective control list to negate those expressed under unstressed conditions, the list of genes common to both the cultivars at 3 h of stress was further modified to obtain a relative fold change (RFC) values by applying the following formula:


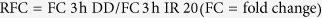


**Figure 2:**
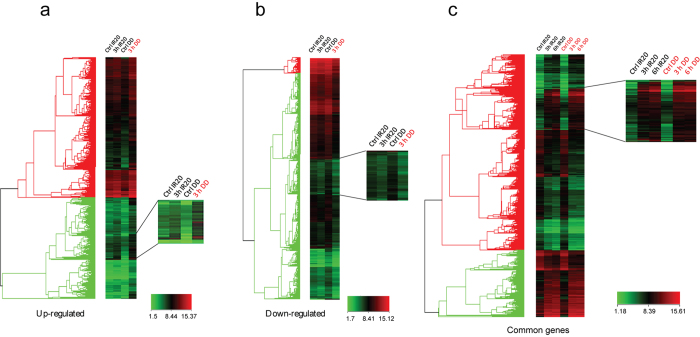
Heat-maps generated for 3 h DD vis-à-vis 3 h IR20. (**a**) Hierarchical clustering of genes up-regulated in DD. The inset shows a cluster of genes up-regulated in DD at 3 h as compared to IR20. (**b**) Cluster of genes down-regulated in 3 h DD. (**c**) Common genes highlighted in both the cultivars. The inset shows the differences in the rate of expression even for the common genes at 3 h. These differences however, diminish at 6 h of stress (third and sixth column from right).

All genes lying between −2 < x > 2 RFC were selected for further analysis. Cluster analysis performed for the generation of heat maps using Hierarchical clustering showed that the rate of change in gene expression was faster for DD than IR20 (Fig. 2). However, these differences are not very discernible at 6 h where the kinetics of DD and IR20 are comparable. The lists of uniquely up/down-regulated genes for each cultivar at 3 h stress were sourced after normalising the signal values obtained in the stressed condition with the expression values of these genes in the unstressed conditions.

### Analysis of the differentially expressing genes under unstressed and stressed conditions

Drought or osmotic stress and salt stress have a complex signalling network that is interconnected to each other. Several studies have been carried out in the past to elucidate the key players and a plethora of genes encoding kinases, DREBs, NAC, WRKY and MYB transcription factors, etc. have been found to play important roles in stress alleviation^25^,^26^. Expression levels of these key genes in DD and IR20 have been listed in Table 1. The drought signalling network as highlighted by ref. 25 is said to be comprised of the Osmotic Stress Signalling (OS), Cell Division and Expansion Regulation Signalling (CDER) and Detoxification Signalling (DS) components. Based on our understanding of the available literature and our own findings, the components of abiotic stress signalling network operating in plants have been pictorially represented in Supplementary Fig. S4. Further, proteins expressed during drought stress were demarcated by ref. 26 into functional and regulatory protein groups. Since several genes are known to be involved in multiple pathways, those highlighted in this study were divided, as much as possible, into separate categories based on previous data and known functions of their orthologs in different species. However, all those genes that could not be confidently placed into any of the defined categories were put in a broader ‘Metabolism’ category. Many DUFs (Domain of unknown function), Pfam-Bs and small peptides (<150 amino acids) were also present. Since these could not be placed in any suitable category, they were treated as outliers and not included for further analysis in the present study. All the categories highlighted in each gene group of DD and IR20 (uniquely up- and down-regulated, as well as common genes) were analysed for their up-regulation or down-regulation. The proportions of the contributions of the various categories were calculated as percentage and stacked graphs were plotted to interpret the results.

**Figure 3:**
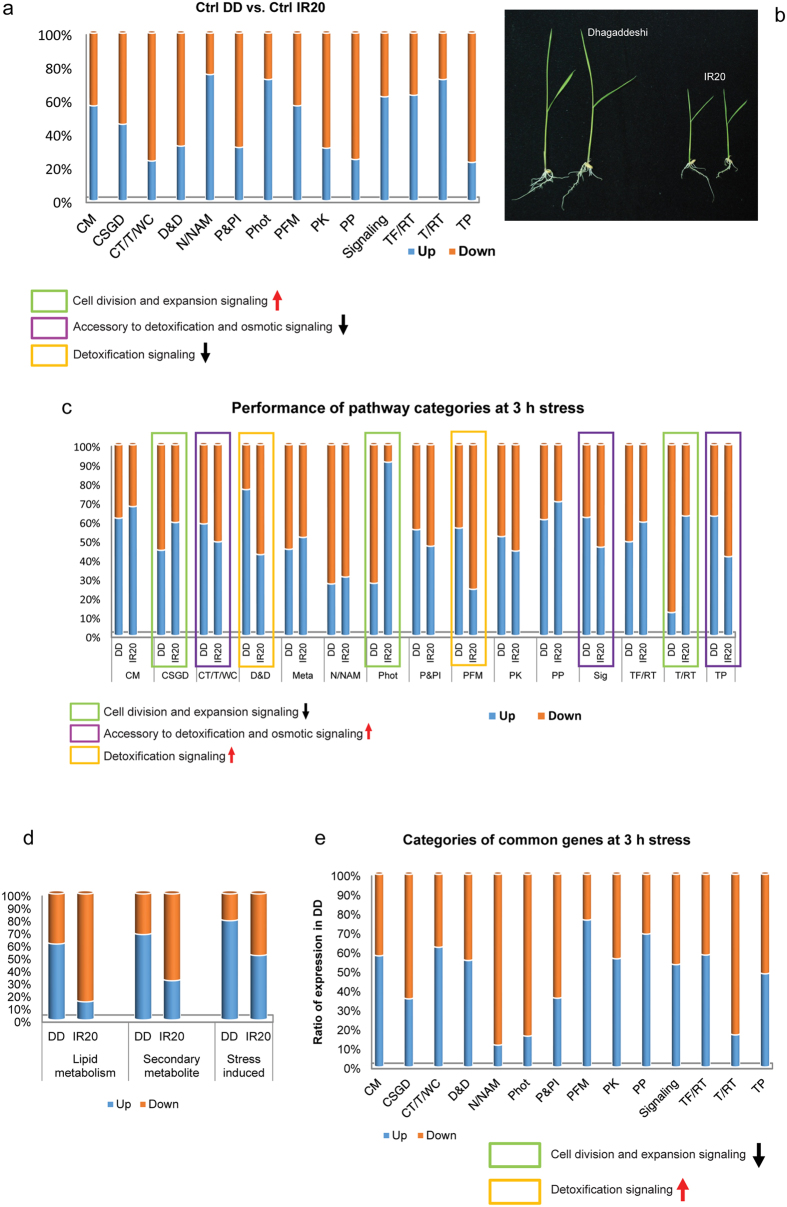
Performance of pathway categories in control conditions and 3 h stress. (**a**) Proportion of the pathway categories at unstressed conditions in DD as compared to IR20. (**b**) Photograph displaying the phenotype of the hydroponically grown 7-day-old seedlings of DD and IR20. The seedlings of DD are considerably taller than IR20. (**c**) Stacked graph of categories in DD and IR20. (**d**) Contributions of accessory categories involved in cell integrity and stress perception. (**e**) Relative expression of common genes in DD and IR20 divided into separate categories. Values indicate the nature of expression in DD as compared to IR20. CM – carbohydrate metabolism, CSGD – cell structure, growth and dynamics, CT/T/W – cellular transport, transporters and water channels, D&D – degradation and detoxification, N/NAM – nucleoproteins/nucleic acid modifiers, Phot – photosynthesis, P&PI – proteases and protease inhibitors, PFM – protection factors of macromolecules, PK – protein kinases, PP – protein phosphatases, Sig – signaling, TF/RT – transcription factors/regulation of transcription. T/RT – translation/regulation of translation, TP – transmembrane proteins.

In the unstressed (control) tissue samples of DD and IR20, the transcript levels of genes belonging to those categories assigned in this study, such as cellular transport/transporters/water channels (CT/T/WC), transmembrane proteins (TP), degradation and detoxification (D/D) and protein phosphatases (PP) were considerably down-regulated in DD as compared to IR20; whereas those pertaining to cell division and expansion regulation (CDER), i.e. nucleoproteins/nucleic acid modifiers (N/NAM), photosynthesis (Phot) and translation/regulation of translation (T/RT) were up-regulated in DD (Fig. 3a). It is known that DD is a taller cultivar compared to IR20 and it was evident even at the 7-day-old two-leaf seedling stage (Fig. 3b). The faster vegetative growth seen in DD could be attributed to the rapid changes taking place in the CDER network that houses genes involved in controlling plant growth. On exposure to stress, however, the expression pattern changed dramatically as compared to the unstressed conditions (Fig. 3c). In DD, among all the genes involved in CDER (green boxes in Fig. 3c) the transcript abundance of many were effectively down-regulated as compared to IR20 whereas the proportion of those involved in Dehydration Signaling (DS) (yellow boxes) and Osmotic Signaling (OS) (purple boxes) were up-regulated in DD. The transcript levels of genes belonging to T/RT category were significantly down regulated in DD, especially those involved in the assembly of ribosomes. This indicated that fresh protein synthesis was probably being arrested in DD as compared to IR20. Up-regulation of the DS category of genes at 3 h stress treatment indicated that the gamut of genes involved in damage control and repair were rapidly activated in DD such that it would be able to achieve homeostasis faster than IR20. Further, the genes involved in lipid metabolism (essential for maintaining cell membrane stability) were also significantly up-regulated in DD vis-à-vis IR20 (Fig. 3d). This also corroborated with the CMS assay (Fig. 1c).

The list of genes (obtained after RFC calculation) depicted the differences in the fold change in the expression of genes that are common to both the cultivars. A similar type of analysis carried out with this group of genes would reveal the differences in the kinetics or rate of gene activation/suppression of the genes for a particular category commonly shared by both DD and IR20. The results plotted in a stacked graph (Fig. 3e) showed that the transcripts of genes representing categories, such as cell structure, growth and dynamics (CSGD), N/NAM, Photosynthesis and T/RT, were significantly down-regulated in DD, whereas protection factors of macromolecules (PFM) was up-regulated. Gene transcripts associated with cell growth and regulation were down-regulated to a greater extent in DD as compared to IR20, including those coding for components of the photosynthetic machinery and the production of new proteins, whereas those involved in protecting the cellular machinery (degradation of unfolded/mis-folded proteins, activation of chaperones, detoxification) were greatly up-regulated in the tolerant cultivar DD. Similar patterns of gene expression as observed in the unstressed conditions (controls) and the common genes obtained after RFC calculation (Fig. 3c,e) could be attributed to the differences in the kinetics of the signaling networks operating in DD vis-à-vis IR20.

### Validation in kinetics of selected genes expressing differentially in DD and IR20 under stress conditions

**Figure 4:**
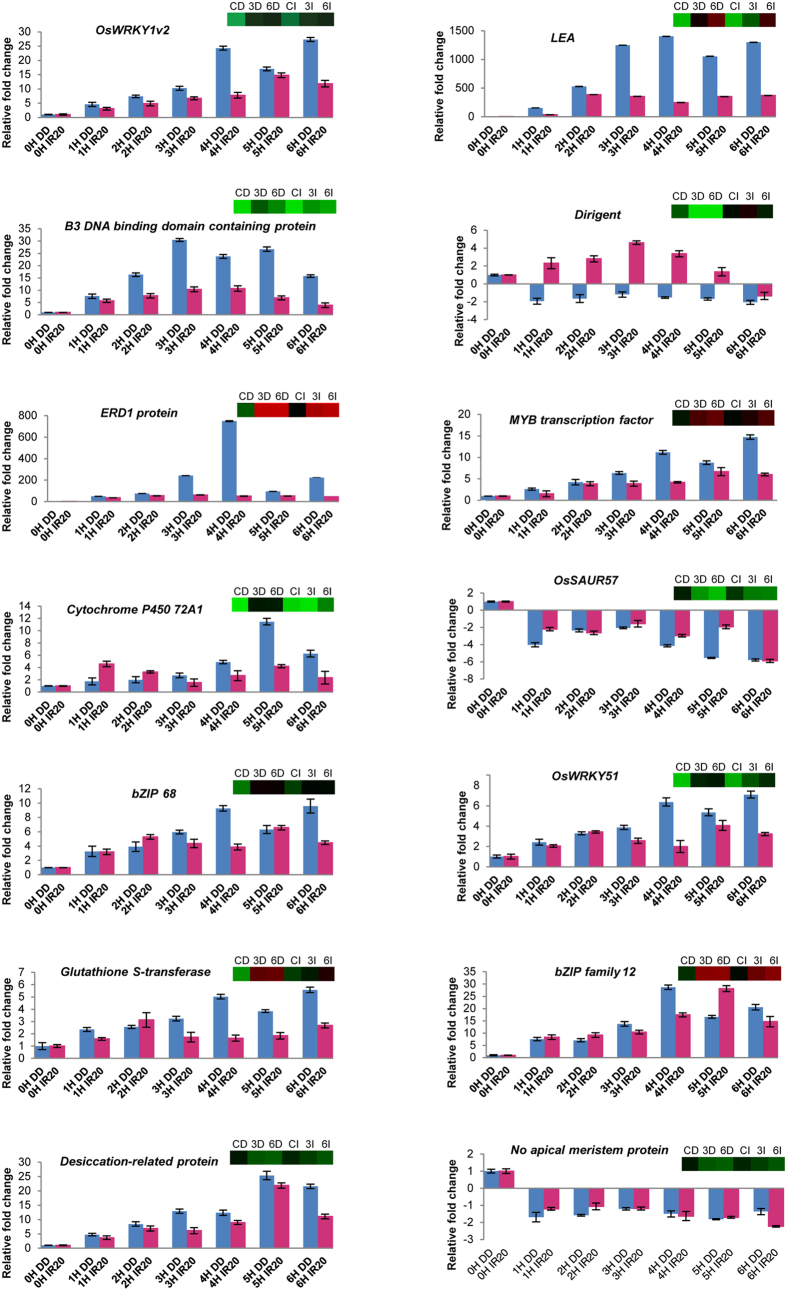
Real time analysis of selected genes in Dhagaddeshi and IR20 over different time points. Hourly intervals of time points were chosen to see the time kinetics of selected genes in respective cultivars. The microarray derived heat-maps of the genes have also been included with each graph (CD: Control DD, 3D: 3 h DD, 6D: 6 h DD, CI: Control IR20, 3I: 3 h IR20, 6I: 6 h IR20).

**Figure 5:**
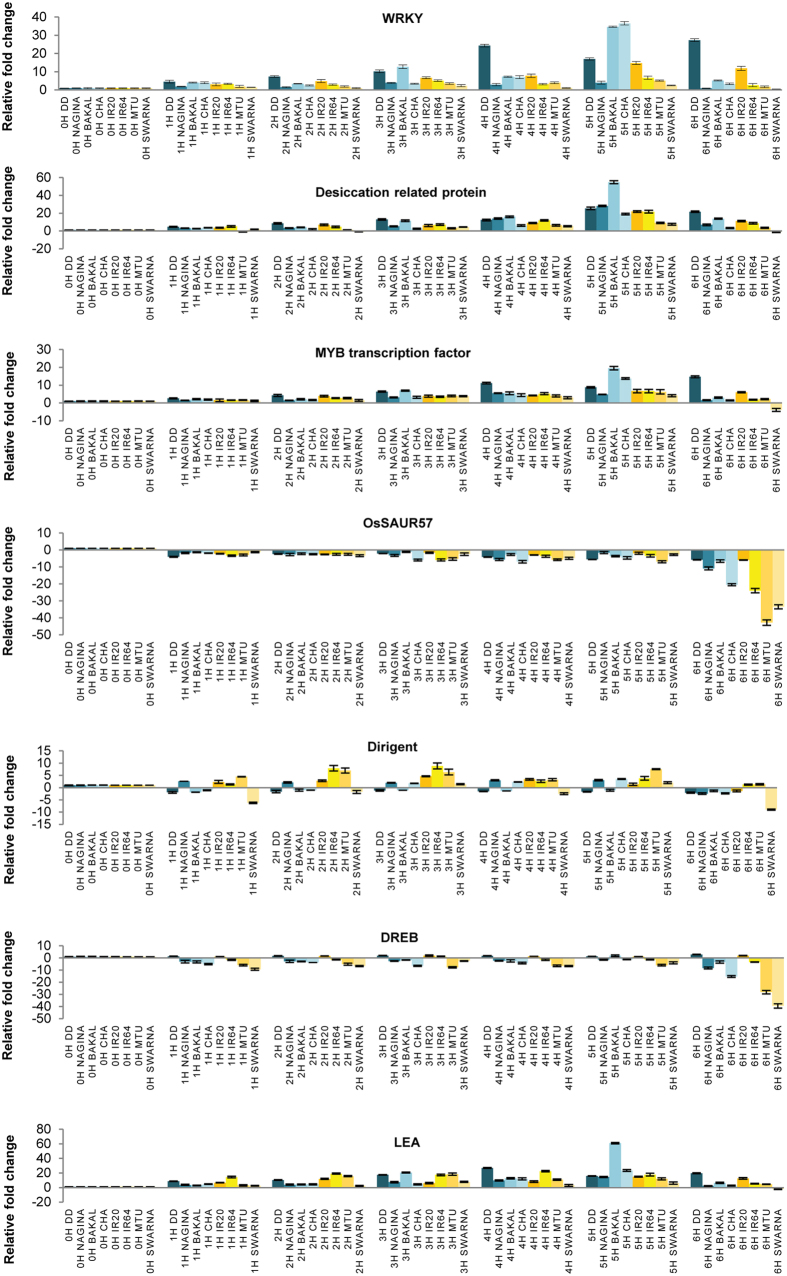
Real time analysis of selected genes over different time points in 8 cultivars. An hourly interval of time points was chosen to see the trend of these selected genes in other cultivars, Dhagaddeshi (DD), Nagina22 (Nagina), Chaptigurmatiya (CHA), Bakal, MTU-1010(MTU), IR20, IR64 and Swarna.

The results of differential gene expression profile generated by microarray were validated using real-time PCR. The genes were selected based on their up/down-regulation and the categories they represent (Supplementary Table S1; Primer sequences: Supplementary Table S2). To elucidate the kinetics of gene expression in DD and IR20 seedlings subjected to drought stress, sampling of tissues in duplicates was carried out on an hourly basis from 1 h to 6 h after stress imposition for both the cultivars. The inclusion of the 1 h and 2 h of stress treatment was purely to observe the expression patterns of the genes selected in response to mild drought stress treatment. Such an exercise would also enable us to identify those genes from the ones selected that respond faster to early drought time periods apart from 3 h of stress, as well as understanding their nature of expression in the contrasting rice cultivars. Real-time PCR data were comparable in terms of changes in gene expression pattern as observed in microarray for nearly all the genes selected for this analysis (Figs 4 and 5). The degree of differential gene expression was observed to be greater in DD than IR20 for most of the genes analysed, for example, *OsWRKY1v2, B3 DNA BINDING DOMAIN CONTAINING PROTEIN (OsVP1), cytochrome P450 72A1, LEA D-34, MYB* and *ERD1*. However, in case of *OsSAUR57*, levels of down-regulation were comparable in DD and IR20 at 6 h of stress; whereas in case of *no apical meristem*, the degree of transcript down-regulation in 6 h IR20 samples was higher than DD. From these real-time data, we could also observe that genes like *OsWRY1v2, LEA, OsVP1, ERD1, MYB, desiccation-related protein* and *OsWRY51* are expressed at a higher level even after 1 h and 2 h of stress treatment in DD than IR20.

A few of the selected genes were checked for their expression in other known drought tolerant (Nagina 22, Chaptigurmatiya, Bakal) and susceptible cultivars of rice (MTU-1010, IR64, Swarna) (Fig. 5). The expression pattern of the selected genes, i.e. *WRKY, MYB transcription factor, desiccation-related protein, OsSAUR57* and *LEA*, in these cultivars was similar to that observed for DD and IR20, respectively, although the levels of expression varied in terms of fold change in these cultivars. However, in case of *dirigent* and *DREB1A*, the pattern of gene expression observed did not conform for DD and IR20. These differences in the expression patterns observed in the various rice cultivars could be attributed to yet unidentified cultivar-specific regulation of gene expression.

### Functional validation of *OsFBK1* in rice and *Arabidopsis*

**Figure 6:**
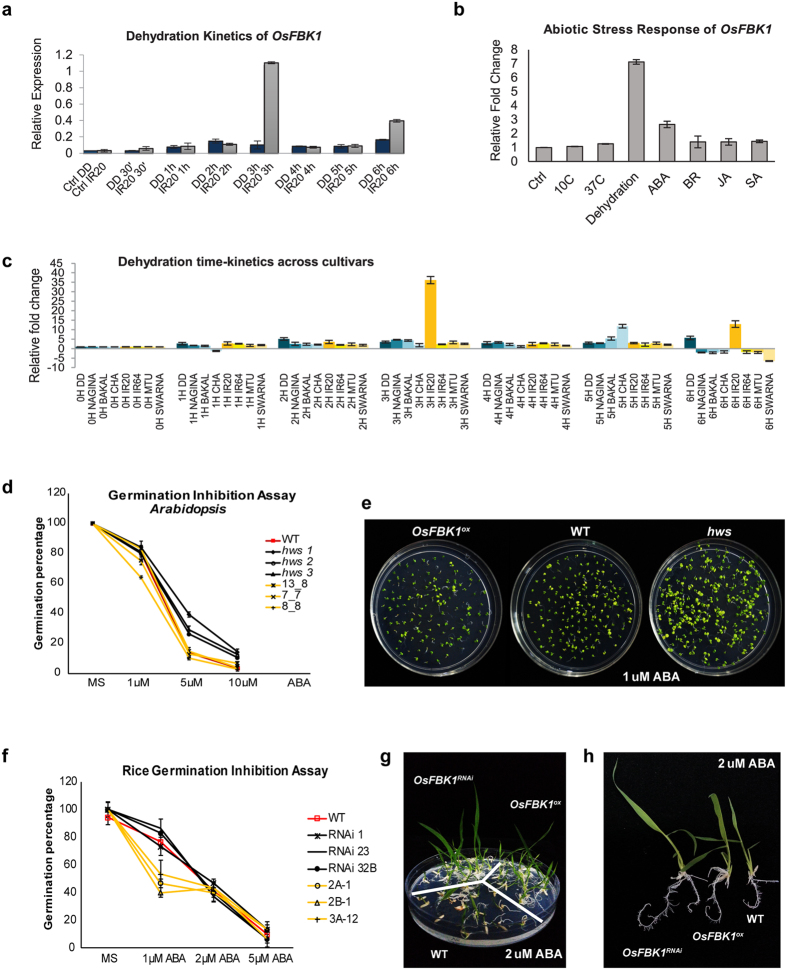
Functional validation of *OsFBK1*. (**a**) Real-time analysis of *OsFBK1* under stress in DD and IR20 at different time points. (**b**) Expression analysis of *OsFBK1* by qPCR under different abiotic stresses. (**c**) Cultivar-specific time kinetics of *OsFBK1.* (**d**) Germination inhibition assay of *Arabidopsis hws and OsFBK1*^*ox*^ lines grown on progressive ABA concentrations. (**e**) Growth differences of the 10-day-old *Arabidopsis* seedlings on 1 μM ABA. (**f**) Graphical representation of germination percentage of rice over-expression lines. (**g**) Differences in the growth rates of 10-day-old rice transgenics vis-à-vis WT on 2 μM ABA. (**h**) Phenotypic differences in the roots of the 10-day-old rice *OsFBK1* transgenics and WT grown on 2 μM ABA.

Although several genes have been found to be expressed more in DD than IR20, one of the genes that was found to be highly specific to IR20 (and hence, of interest) during stress imposition was *OsFBK1*, an F-box protein encoding gene that is a part of the degradation and detoxification (D/D) category (Fig. 6a and c). This uncharacterized gene was first identified by our group^21^ and was found to be expressed more under dehydration stress, and also in response to externally applied ABA (Fig. 6b). The protein sequence of OsFBK1 was found to be highly conserved in both *japonica* and *indica* species of rice (Borah and Khurana, unpublished). *OsFBK1* has a close relative in *Arabidopsis* known as *HAWAIIAN SKIRT*^27^*(HWS)*, although its role in stress is not yet explored. For functional validation, over-expression transgenics of *OsFBK1 (OsFBK1*^*ox*^) were raised in both *Arabidopsis* and Pusa Basmati 1 (PB1) cultivar of rice (drought susceptible), to draw parallels (if any) between its role in both dicots and monocots in imparting stress response. Knock-down lines (*OsFBK1*^*RNA*i^) were also generated in PB1 using the RNAi-mediated approach. Apart from demonstrating several phenotypic changes (Borah and Khurana, unpublished), the transgenics, displayed drought tolerance when grown on ABA supplemented medium (Fig. 6e and g). However, the *hws* mutant of *Arabidopsis* and the *OsFBK1*^*RNAi*^ transgenics performed better than the over-expression transgenics in terms of germination on ABA enriched MS medium (Fig. 6d and f). It was also observed that while the growth of the over-expression transgenics lines in both species on prolonged ABA exposure were comparable to WT, the mutant/knock-down lines fared exceedingly well than both WT and over-expression transgenics (Fig. 6e and g). The knock-down rice transgenic seedlings also had better root growth than WT on exposure to ABA in terms of root hairs indicating stress alleviation (Fig. 6h). Interestingly, the over-expression lines also displayed slight better root growth than WT (Fig. 6h). Real-time PCR and protein estimation of OsFBK1 in the rice transgenics were also carried out to confirm the expression of the gene in the transgenics (Borah and Khurana, unpublished), where it was proved that the over-expression and knock-down lines were true lines and not escapes. These experiments suggest that silencing *OsFBK1* would prove to be a better strategy than over-expressing it in susceptible cultivars of rice and thus, could be a candidate gene for providing drought tolerance to rice. A more detailed experimentation is however required to firmly establish the role of this gene in conferring drought tolerance.

Apart from *OsFBK1*, we are also investigating several other genes mined from our analyses; however, it is beyond the scope of the present study.

### Distinct and unique metabolic pathways are regulated in DD under drought stress

**Figure 7:**
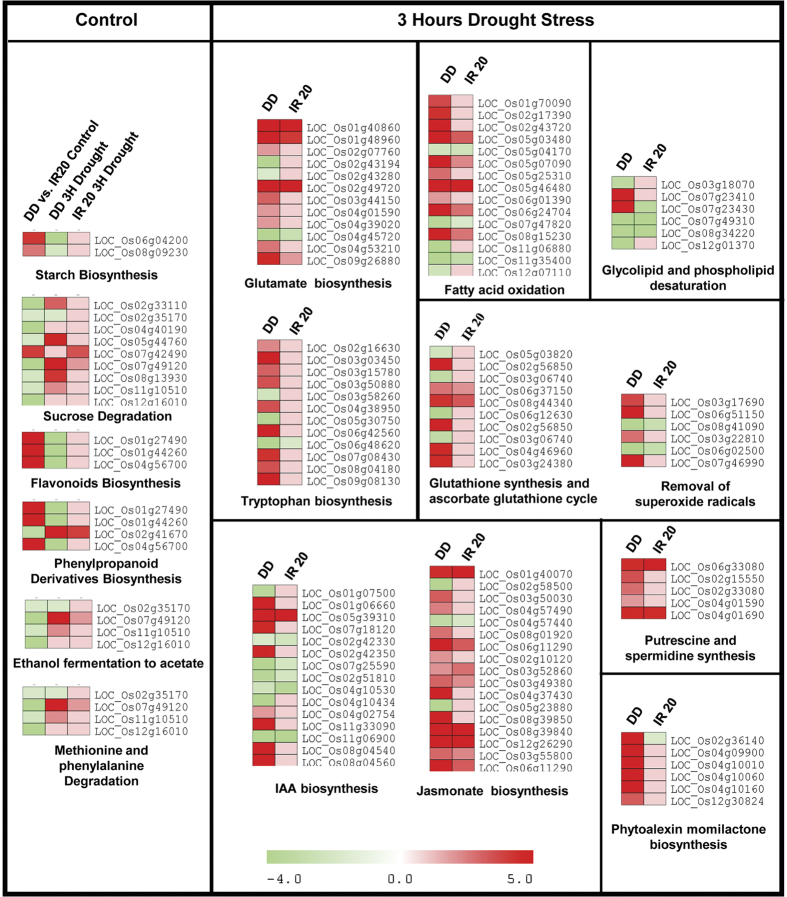
Regulation of metabolic pathways during drought stress. The metabolic pathways enriched in differentially expressed genes of Dhagaddeshi under 3 h drought stress are shown with heat-maps representing their expression profile. The scale represents log_2_ fold change in expression.

To generate the metabolic profile based on the differentially expressed genes under water deficit conditions, the microarray expression data were integrated with metabolic pathways available at Gramene RiceCyc database (version 3.3). Under control conditions, the pathways involved in biosynthesis of secondary metabolites such as phenypropanoid derivatives, carbohydrate metabolism and degradation of amino acids were significantly enriched among the differentially expressed genes (Fig. 7, Enrichment analysis, Fisher Exact test, p < 0.1). The transcript levels of gene encoding *leucoanthocyanidin dioxygenase* (LDOX) were high in DD under control conditions which is a key enzyme for anthocyanin and proanthocyanidin production in *Arabidopsis*^28^,^29^.

**Figure 8:**
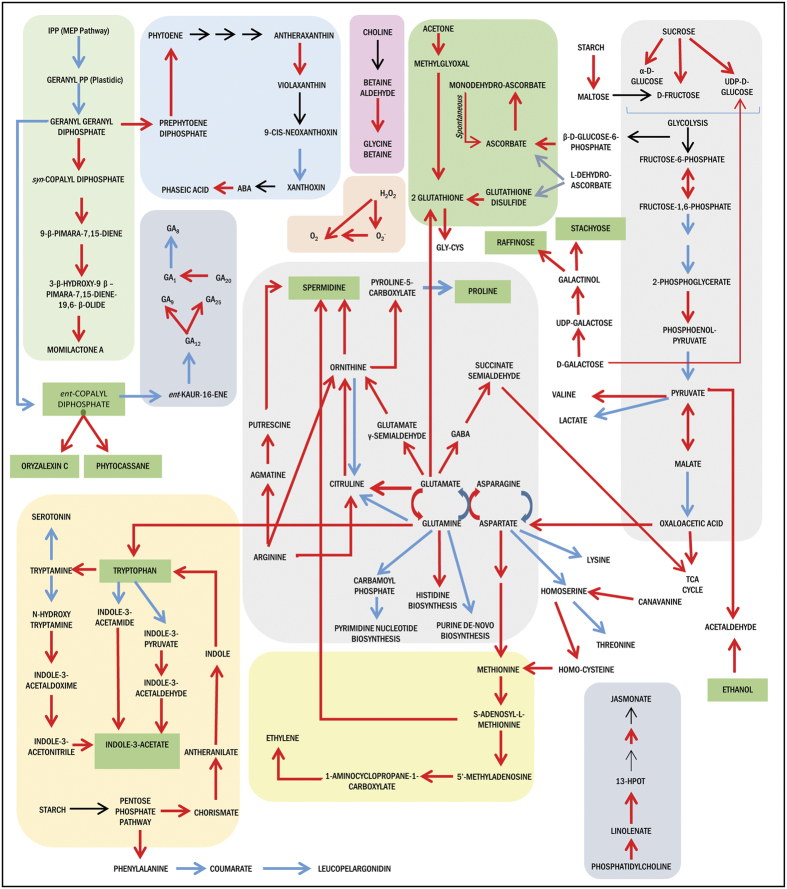
Schematic representation of major metabolic pathways regulated under drought stress. The metabolic pathways enriched in differentially expressed genes of Dhagaddeshi under 3 h drought stress were reconstructed using Gramene RiceCyc database. Arrows highlighted in Red, Blue or Black represents the expression level of gene loci, i.e. Up-regulated, Down-regulated or unchanged/not represented respectively.

The pathways involved in amino acid metabolism, hormone biosynthesis, redox homeostasis, and secondary metabolite biosynthesis were found to be significantly enriched in the analysis carried out based on DEGs from DD after 3 h of stress (Figs 7 and 8; Supplementary Fig. S5, Supplementary data S1). Based on the nature of the pathways highlighted and the interactions existing amongst them, a schematic representation of the major metabolic pathways regulated under drought stress has been generated (Fig. 8). Number of metabolic pathways found to be common between the two cultivars include biosynthesis of ABA, jasmonate, polyamines, stachyose and proline. However, pathways such as biosynthesis of tryptophan, fructans, phenylpropanoids, diterpenoids, momilactone, oryzalexin C, phytocassane, chalcones and glutathione redox pathways were uniquely affected in only DD seedlings subjected to 3 h of dehydration stress; also, transcript abundance of genes associated with gluconeogenesis, citrulline and phytocassane biosynthesis were up-regulated in DD but down-regulated in IR20. Transcripts of enzymes involved in metabolism of amino acids such as tryptophan, histidine, ornithine and γ-aminobutyric acid (GABA) were found to be up-regulated whereas glutamate, lysine and homoserine were down-regulated in DD under stress. The transcript levels of genes associated with biosynthesis of antioxidant metabolites, glutathione and ascorbate along with enzymes such as glutathione peroxidase, ascorbate peroxidase 1, catalase isozyme B, cytosolic Cu/Zn-superoxide dismutase, glutathione reductase and monodehydroascorbate reductase were also high in DD seedlings under stress. However, the transcript levels of these genes involved in redox homeostasis were slightly reduced or remained unchanged in IR20 under stress. Also, the genes encoding factors involved in methylglyoxal degradation pathway to glutathione were found to be induced more strongly in DD than IR20, thereby contributing towards detoxification of methylglyoxal to glutathione more effectively in DD than in IR20. Methylglyoxal, a potent reactive cytotoxic compound produced in plants under various stressful conditions, is a common phenomenon, and it could therefore act as a signal for plants to respond to stress^30^. It is detoxified via the glyoxalase system that involves glutathione^31^,^32^.

Among osmolytes, down-regulation of transcript level of the gene encoding proline dehydrogenase, involved in proline degradation, was observed that could lead to decreased catabolism of proline. Our analysis also highlighted genes associated with pathways involved in biosynthesis of glycine betaine, fructose, sucrose, stachyose and raffinose under dehydration in DD. The transcript levels of the gene encoding *phenylalanine ammonia-lyase* (PAL) were down-regulated under control conditions but observed to be higher in DD than IR20 after 3 h of stress. As shown in Fig. 7, genes involved in metabolism of indole derivatives were also highlighted in DD under stress that could lead to increased production of plant hormone auxin (indole-3-acetic acid). Among other hormones, genes in discrete steps involved in metabolism of ABA, jasmonate, ethylene, GA and polyamines, such as putrescine and spermidine, were also differentially expressed. Although the transcript levels of genes involved in ABA biosynthetic pathway were similarly induced in both the cultivars, the induction of genes involved in jasmonate, ethylene, putrescine and auxin synthesis was much higher in DD than IR20 under stress. Other pathways that were found to be enriched based on the analysis carried out with differentially expressed genes included fatty acid oxidation, glycolipid and phospholipid desaturation that may play a role in stability of membranes under dehydration stress as observed in physiological assays where DD showed reduced ion leakage under stress.

### Co-localization of DEGs and overlapping QTLs on rice chromosomes

**Figure 9:**
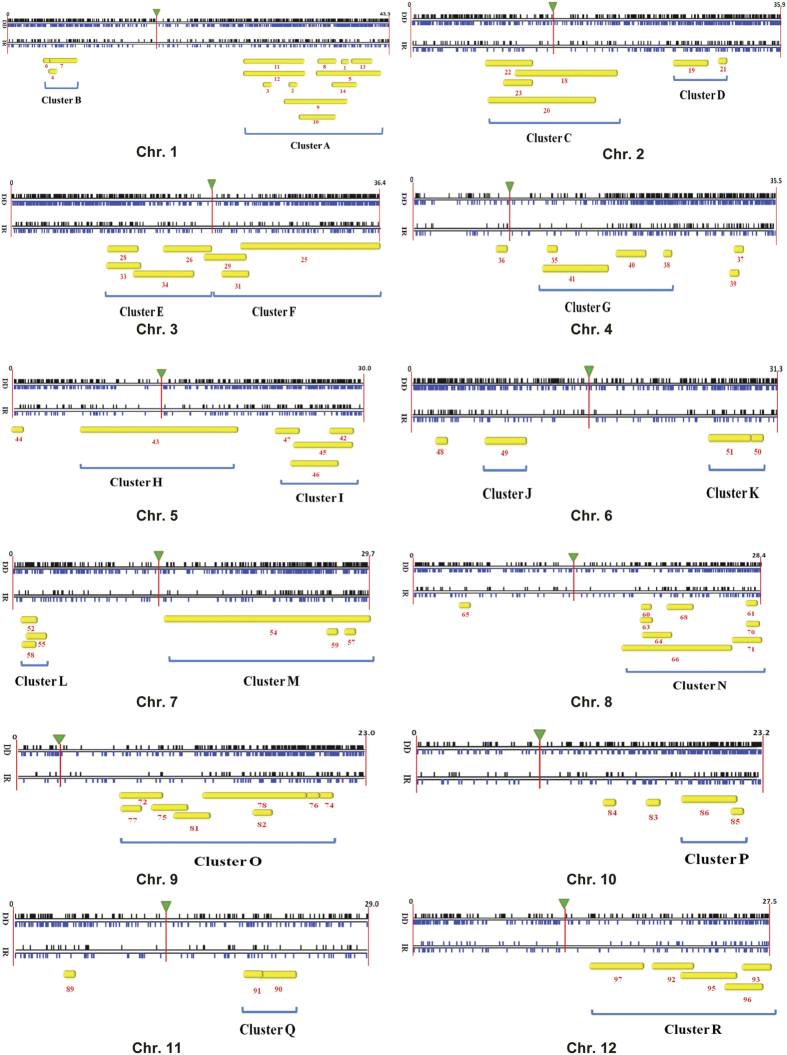
Chromosomal localization of differentially expressed genes and overlapping drought responsive QTLs on rice chromosomes. Black and Blue vertical dashes represent up-regulated and down-regulated genes (after 3 h drought stress) on chromosomes, respectively. Inverted green triangle represents the position of the centromere. Yellow bar represents the position of overlapping QTL with the chromosome. (DD: Dhagaddeshi, IR: IR 20)

**Figure 10:**
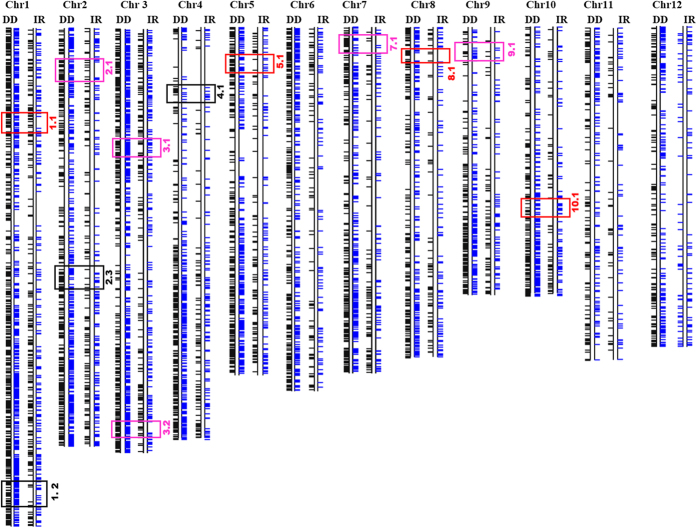
Distribution of DEGs from Dhagaddeshi and IR 20 after 3 h of drought stress on rice chromosomes. Horizontal dash indicates a gene positioned to its known physical position on chromosome. Black and Blue horizontal dashes represent up-regulated and down-regulated genes (after 3 h of drought stress) on chromosomes, respectively. The identified genomic blocks with a difference in expression pattern between the two cultivars are highlighted in red (significantly up and down-regulated), black (significantly up-regulated only) and pink (significantly down-regulated only) for p-value < 0.05.

Drought tolerance is a complex and quantitative trait with large number of genes each with small to medium effects controlling the outcome. Previous studies have identified several Quantitative Trait Loci (QTLs) for drought tolerance, osmotic adjustment capacity, cell membrane stability, ABA content among other drought related secondary traits. We could identify 97 QTLs from Gramene database, Tropgene database and previous studies^5^,^9^,^33^,^34^ pertaining to observed physiological traits of the contrasting rice cultivars in this study (Supplementary Table S3). All DEGs were distributed on the chromosomes based on their known positions on the physical genome of rice and, by alignment of QTLs based on their chromosomal coordinates, stress responsive genes underlying these QTLs were determined (Fig. 9). Further, by localization of DEGs to their known positions on the physical map of the rice genome, we could identify 11 genomic blocks with significant differential gene expression between DD and IR20 (Fig. 10; p < 0.05). Four of these blocks enriched in DEGs [named as 1.2 (chr1: 39270532–40943109), 3.2 (chr3: 33731404–34972748), 7.1 (chr7: 664612–2184998) and 10.1 (chr10: 14678069–16149965)] co-localized with QTLs identified previously for drought tolerance and cell membrane stability (Fig. 10). Block 1.2 localized to the region under QTL no. 13, *qDTY1.1* (chr1:38895261–40580568), for grain yield under drought tolerance in DD. Block 3.2 co-localized with QTL for drought tolerance (AQHP069, chr3: 22798284–35828040). Block 7.1 co-localized with QTL for cell membrane stability (DQA3/QCMS7.1, chr7:1160982–1537879) and osmotic adjustment capacity (Figs 9 and 10). Notably, both DD and IR20 cultivars were observed to have differential cell membrane stability after 3 h of stress (Fig. 1C).

Several of the QTLs selected in this study were found to overlap with each other when positioned on the rice chromosomes. Thus, for the fact that they appear as a unit, such regions on the chromosomes could be grouped into 18 clusters (Cluster A-R) (Fig. 9; Supplementary data S2). The distribution of genes underlying these clusters was observed to be non-random in terms of their functional categories. Most of the clusters were populated with ‘Metabolism’ related genes which were either up- or down-regulated, thereby making it the largest category in the clusters (Supplementary data S2). Cluster L and R contain 26% and 11%, respectively, of ‘Stress Induced’ genes, which were uniquely up-regulated at 3 h of stress in DD vs. IR20. A greater percentage of genes which are involved in cell structure, growth and dynamics (CSGD) were down-regulated in DD w.r.t. IR20 in many of these stress clusters, viz. G, H and J. Apart from this, a category of genes functioning in Degradation and Detoxification (D/D) was uniquely up-regulated in a number of clusters and maximum in Cluster B and P: 22% and 28%, respectively. Also, the genes involved in CT/T/WC and Signalling (Sig) were up-regulated in DD whereas those involved in N/NAM and T/RT were down-regulated in significant numbers. There were a large number of genes which were present in these clusters that could not be functionally categorised and these were classified as Unknown category.

## Discussion

The Indian subcontinent is a huge repository to several indigenous cultivars of rice that are naturally endowed to overcome abiotic/biotic stresses. However, these cultivars also suffer from possessing the undesirable trait of being low-yielding, whereas the stress-susceptible ones are high-yielding. Several studies have been carried out in N22 and IR64 cultivars and transcriptomic data on them are also available in public databases. However, other indigenous cultivars are less explored. Hence, to obtain a better perspective of the molecular machinery operating in such unexplored cultivars and comprehensively analyse the stress associated metabolic network, we performed comparative transcriptome analysis of Dhagaddeshi and IR20 rice cultivars by microarray under control and drought stress conditions at the seedling stage.

First, in order to carry out an in-depth analysis of the nature of the pathways operating under drought stress in these cultivars, the GO categories were further refined in order to incorporate the signalling networks identified previously^25^,^26^. Our microarray analysis revealed that the expression of genes encoding components involved in DS and CDER sub-networks were distinctly altered in the contrasting cultivars during 3 h of drought stress. The components of the DS network were activated earlier in Dhagaddeshi than in IR20 and, likewise, the machinery controlling cell growth and expansion was arrested faster in DD as compared to IR20 in response to the imposed dehydration stress (Figs 3, 4 and 7). At molecular level, DD responded to dehydration by increasing the levels of transcripts encoding for proteins, such as LEA, DREBs, NACs; enzymes for carbohydrate metabolism and ROS scavenging antioxidants (Table 1). The assumptions made in this respect were substantiated by a more detailed kinetic analysis of differential gene expression, as revealed by qPCR analysis (Figs 4 and 5).

It proposed that under salt and drought stress conditions, disturbances in the ionic and osmotic equilibrium activate the networks that work to bring about ionic and osmotic homeostasis in the plant resulting in stress tolerance^25^ (Supplementary Fig. S4). At the same time, injuries resulting due to water stress also activate the DS and CDER networks. While the activation of the CDER results in growth inhibition, the DS network brings about damage control and repair. All these networks work closely with each other to alleviate stress and due to the sheer complexities in their functioning they have considerable number of overlapping regulators, making it extremely difficult to dissect the key players unambiguously. Nevertheless, in our data the alteration in the expression of genes encoding components of the detoxification network was fairly identifiable at 3 h stress (Figs 3 and 4). However, as both the cultivars underwent more than 70% loss in their water content after the imposition of 6 h of drought stress (Figs 1 and 2), the expression data obtained at this time point was comparable for both the cultivars, to a large extent. Thus, it was prudent not to include the data derived from this time point. The seedlings of DD were thus found to be able to sense drought stress faster than IR20 and respond to it by altering the expression of genes that are involved in the regulation of growth and at the same time activating the osmotic and detoxification signaling networks to achieve tolerance against the prevailing drought stress much before IR20. These data corroborate the observations made earlier by^19^, where they found that genes partaking in cell wall remodelling and antioxidant defence system were up-regulated in the tolerant cultivars of N22 and Pokkali as opposed to IR64 (susceptible). *EARLY RESPONSIVE TO DEHYDRATION (ERD1),* which is known to be rapidly activated under drought stress was preferentially expressed at high levels in DD only and substantiates our hypothesis that DD seedlings sense and respond faster when exposed to dehydration stress (Fig. 4, Table 1). DD was found to accumulate anthocyanins (observed phenotypically) even under control conditions (data not shown). Unstressed DD seedlings showed increased transcript levels of *Leucoanthocyanidin dioxygenase (LDOX*), a key gene for anthocyanin and proanthocyanidin production in *Arabidopsis* (Fig. 7). Anthocyanins have been reported to accumulate under drought and cold stress and such plants have been shown to be more resistant to drought^35^. This provides support to assumption that Dhagaddeshi has inherent preparedness to counter drought conditions and thereby respond quickly^29^,^36^.

As highlighted in Table 1, number of previously identified drought stress related genes were shared by both the tolerant and susceptible cultivars, however, their expression levels varied considerably. Our data corroborate previous reports on the involvement of genes encoding TFs, such as *OsDREB, OsLEA, OsWRKY, OsMYB* and *OsbZIP*, in abiotic stress response, and it could also be validated using real-time PCR analyses (Figs 4 and 5). The expression of genes, such as *OsDREB2A, OsDREB2B, OsLEA3-1, OsLEA4, SNAC1, SNAC2, OsNAC4, OsNAC10, OsbZIP12*, was considerably higher in DD than IR20 at 3 h water deficit stress. *OsFBK1*, the previously uncharacterised component of the D/D signalling pathway was found to be specific to IR20, and knock-down transgenics of *OsFBK1* were found to impart tolerance against prolonged ABA exposure vis-à-vis untransformed plants. Although the over-expression plants were also found to impart some tolerance, the knock-down lines or mutant lines fared much better. Such same responses of over-expression and loss-of-function lines have been previously reported in several other non-stress studies also^37^. The transgenics of *OsFBK1* have also been found to demonstrate several phenotypic changes and the data have been arranged in a separate manuscript (Borah and Khurana, unpublished). Transgenic rice plants overexpressing *OsDREB2A* and *OsDREB2B* have been found to increase drought tolerance and show increased content of soluble sugars and free proline at seedling stage^38^,^39^. In our data, transcripts levels of genes involved in synthesis of soluble sugars, raffinose and stachyose, were observed to increase in both the cultivars (Supplementary Fig. S5). Similarly, accumulation of free proline under water-deficit was found to be similar in both cultivars. LEA proteins also act as stabilizers of proteins and membrane^20^. Under stress, seedlings of DD were found to show higher membrane stability or low ionic leakage as compared to IR20 (Fig. 1c). Notably, *OsLEA3-1* cDNA has been found to be induced by ABA in a cultivar specific manner and its over-expression in rice improves drought resistance^40^,^41^. Expression levels of *OsLEA3-1* (FC = 779.7 vs control) and *OsLEA4* (FC = 258.7 vs control) were also found to be much higher in DD. The enhanced expression of *OsNAC2, OsVP1, OsABI5/OsABF1/OsbZIP10, DRO1* and *OSISAP1* was exclusive to DD only. Thus, it appears that faster response towards stress by DD is a cultivar-specific mechanism that may be responsible for a unique regulation of signalling and metabolic pathways in DD that otherwise may be differently regulated in IR20, a fact exemplified by the dehydration kinetics of the IR20-specific gene, *OsFBK1* (Fig. 6). The functional validation of *OsFBK1* in a susceptible cultivar, PB1, also shows that while IR20 also houses genes whose expression can be modulated to confer stress tolerance, the total number of such genes regulated under duress is phenomenally lesser than the tolerant DD, which in turn does not confer IR20 with the requisite mechanisms to combat stress conditions. Thus, transcriptome studies such as ours could serve as a guide for down-stream experimentations and applications for generating stress-tolerant cultivars for commercial uses, as users could use such information to make appropriate choices while selecting candidate genes from either cultivar.

Most, but not all, of the dehydration inducible genes are also induced by the phytohormone ABA^42^. Dissection of ABA regulated responses is thus essential towards elucidation of signalling networks operating in DD under stress. NCED, 9-cis-epoxycarotenoid dioxygenase, is the key enzyme for ABA biosynthesis in plants. Five genes, *OsNCED1 to 5*, have been identified in rice. In our analysis, genes for ABA biosynthesis were induced in both the cultivars under dehydration (Supplementary Fig. S5). However, transcript levels (after normalization with respective control tissues) for *OsNCED4* (LOC_Os07g05940), involved in ABA biosynthesis, were almost double in DD (>600 FC Vs Control) than IR20 (>300 FC Vs control). *OsNCED4* transcripts were found to be strongly up-regulated by salt stress^43^. In contrast to other ABA biosynthesis genes, *OsNCED5* (LOC_Os12g42280) showed >3 Relative FC increase in IR20 (>100 FC vs control) as compared to DD (Supplementary Fig. S5). *OsNCED5* has been reported to act as osmotic sensor and is also induced by high glucose concentrations^44^. Accumulation of glucose, fructose and sucrose has also been observed under dehydration stress^45^. We also observed up-regulation of enzyme coding genes for starch degradation, sucrose and glucose synthesis in our analysis. In addition, levels of *OsNCED1* (LOC_Os02g47510) were found to be uniquely down-regulated in DD. Further, the transcript levels of Os*ABA8ox1* (LOC_Os02g47470) involved in ABA inactivation were also found to be higher in DD than IR20. The breakdown of ABA catalysed by *OsABA8ox1* and the resulting lower levels of ABA could be advantageous to plants under prolonged drought stress^46^. Activation of this gene may also contribute in part to reduction in ROS levels^47^. The transcript levels of *OsNCED* genes were thus concurrent with the estimated total ABA content that was higher at 3 h stress and declined thereafter and stabilized (Fig. 1d). It also explains a steady rise in ABA levels in DD seedlings under stress compared to a rapid increase in ABA levels in IR20 (Fig. 1d). Considering the magnitude of differentially expressing genes and expression levels, it appears ABA independent pathways of drought signalling networks are also regulated uniquely in DD. Much higher transcript levels of genes directly modulated by ABA levels, such as *OsLEA3-1, OsLEA4, OsLEA1a*, along with indirectly regulated *OsDREB* genes in DD, lead us to believe that stronger regulation of both ABA dependent and independent mechanisms of drought stress tolerance might operate in DD, whereas only ABA dependent pathways might be predominantly regulated in IR20 at least during early stages of drought stress imposition, as per our analyses.

Dehydration stress enhances production of ROS and ROS-associated peroxidation reactions leading to damage of cellular structures^48^. Being essential for cellular signalling, maintenance of ROS level depends on the balance between ROS production and scavenging. Thus, the kinetics of ROS detoxification reflects the ability of the tissue to acclimate the energy imbalance caused due to rising ROS levels^49^. Analysis of genome scale metabolic pathways in DD revealed up-regulation of genes involved in biosynthesis of antioxidant enzymes and metabolites (Figs 7 and 8; Supplementary Fig. S5). Metabolic pathways such as glutathione dependent redox reactions, ascorbate glutathione cycle and genes involved in removal of superoxide radicals were uniquely expressed at higher level in DD under dehydration stress at 3 h. Many phytoalexins, such as momilactone, oryzalexin and phytocassanes, may also quench ROS that could explain their synthesis under different biotic and abiotic stresses^50^,^51^. Increased levels of *zeaxantin epoxidase 1* (LOC_Os04g37619) and reduced *violaxanthin de-epoxidase* (LOC_Os04g31040) are instrumental in the regulation of xanthophyll cycle and maintenance of redox homeostasis in plants^52^,^53^. The changes in transcript levels of these genes also indicate towards increase in reduced ascorbate levels and faster dissipation of reducing equivalents, NADPH, thus, favouring maintenance of lower oxidative stress levels in DD. Increased content of sugars such as glucose also contribute to synthesis of the antioxidant metabolite ascorbate leading to ROS quenching (Fig. 8). Sugars may also directly scavenge ROS^54^. Tryptophan biosynthesis pathway is found to be induced under amino acid starvation, oxidative and abiotic stress conditions^55^. GABA metabolism has also been associated with carbon-nitrogen balance, ROS scavenging and stress tolerance^56^,^57^. Many WRKY family TFs have been shown to be modulated by salicylic acid (SA), jasmonic acid (JA), ABA and provide resistance towards bacterial diseases in rice by stimulating production of PR proteins and above mentioned phytoalexins, such as momilactones, oryzalexin and phytocassanes^58^,^59^. In our analysis, 14 WRKY TF encoding genes were uniquely upregulated in DD compared to only 2 in IR20, whereas 8 such genes were commonly regulated between both cultivars. Few of these were chosen and validated by real time PCR analysis at different time points that confirmed their increased expression in drought tolerant cultivars (Figs 4 and 5). This class of TFs is also known for their role in linking biotic and abiotic stresses and, thus, it also hints at the probability of the ROS-mediated redox pathways playing a crucial role in DD’s tolerance towards drought stress as compared to IR20.

As mentioned earlier, the components of detoxification signalling were activated earlier in DD than IR20. These involved antioxidant defence responses including glutathione as conjugate and a redox metabolite. In our analysis, number of glutathione-S-transferases were uniquely regulated in DD specifically in the Detoxification Signalling category. This amounted to 28 (8.3%) up-regulated, 11 (10%) down-regulated and 7 (5.6%) common GST encoding genes in DD or common between both cultivars, respectively. It is worth noting that these GSTs can be modulated by abiotic stresses, ABA, JA and auxin^60^. The over-expression of GST encoding genes has also been found to increase stress adaptability in transgenic tobacco and *Arabidopsis* plants^61^,^62^. Interestingly, apart from GSTs, transcripts of a number of auxin responsive genes (Log_2_ FC > 10, RiceXPro Version 3.0) were uniquely up-regulated in DD whereas majority of the ABA responsive genes were expressed similarly between both cultivars. Pathway analysis also identified number of genes associated with indole and auxin metabolism (Figs 7 and 8). Although the role of auxin under abiotic stresses remains unclear, functional genomic studies have provided cues towards its complex role that may involve crosstalk with ROS and redox pathways^63^. In fact, oxidative stress can induce a broad spectrum auxin–like effects on seedlings in *Arabidopsis* and these effects involve changes in auxin distribution and content^64^.

A crucial detoxification pathway mediated through glutathione is the methylglyoxal pathway that is an offshoot of glycolysis where glucose is converted to methylglyoxal and then to pyruvate. Methylglyoxal production does not lead to the generation of ATP and it is a cytotoxic compound that is ubiquitously removed by the glyoxalase pathway. This detoxifying pathway comprises of two enzymes that first utilises glutathione to produce an intermediary and then regenerates it in the subsequent reaction^65^. In our study, methylglyoxal pathway related genes were distinctly upregulated in DD as compared to IR20 indicating that the detoxification pathway was activated earlier and preferentially in the tolerant cultivar (Fig. 8). Thus, it can be suggested that crosstalk between ABA, JA, auxin and redox pathways forms the first line of defence in Dhagaddeshi, with ROS as input signals activating osmotic stress and detoxification signalling.

There is a growing body of thought that claims the presence of stress-induced chromatin alterations that are heritable and are transmitted to their progenies, resulting in desirable characteristics^66^. In addition, there are a number of reports on chromatin modification upon external stimuli and, among abiotic stress factors, the epigenetic deregulation and transposon activation by heat stress has been best documented^67^,^68^,^69^. The presence of gene clusters along specific regions of the chromosomes as highlighted in our present study might indicate to the possibility of differential regulation of chromatin under different stress conditions leading to such a differential expression among two cultivars (Figs 9 and 10). Drought tolerance is a quantitative trait and several earlier studies have identified QTLs for drought tolerance in rice. However, only few have been characterised for genes underlying these QTLs. Thus, it is imperative to identify novel genes for drought tolerance. In this regard*, DEEPER ROOTING 1 (DRO1)* gene cloned from DRO1 QTL was found to increase root angle thereby leading to high yield under drought conditions in ref. 70. We found that the expression of *DRO1* gene (LOC_Os09g26840) was uniquely upregulated in DD only after 3 h dehydration stress. To mine stress responsive genes underlying QTLs, we correlated known QTLs with expression analysis to identify potential genes for drought tolerance (Fig. 9). Further, by localization of DEGs to their known physical positions on different chromosomes, a number of genomic blocks were identified (Fig. 10). The distribution of DEGs with reference to their known functions appeared to be non-random, with few chromosomal regions showing more even distribution of genes with diverse functions whereas few others were enriched in genes belonging to particular functional categories. For example, in DD, clusters B and P were populated more with the genes categorized in the Degradation/Detoxification group, whereas the genes functioning under stress induction were highlighted more in clusters L and R (Fig. 9).

In our analysis, co-localization of genomic blocks of differential expression with the known QTLs identified certain regions on chromosomes that show more vulnerable expression profiles under stress. A major effect QTL, *qDTY1.1* (chr1:38895261–40580568), from Dhagaddeshi linked to grain yield under drought was identified recently^9^. It was interesting to observe that the genes underlying *qDTY1.1* (QTL number 13 in our data) showed significant differential expression and also co-localized to genomic block 1.2 (Figs 9 and 10). Few genes such as *FRUCTOSE-BISPHOSPHATE ALDOLASE* (LOC_Os01g67860), *OsVP1* (LOC_Os01g68370), *AUXIN RESPONSE FACTOR 2* (LOC_Os01g70270) showed high expression levels along with other conserved genes and those of unknown function (see supplementary file for list of DEGs under *qDTY*_*1.1*_). *Fructose-bisphosphate aldolase* (FBA) is a key enzyme for glycolysis, gluconeogenesis as well as Calvin cycle. Interestingly, FBA activity is known to increase in response to GA and drought stress^71^,^72^. Further, a tight linkage between *sd1* allele and *qDTY*_*1.1*_ in tall landraces most of which are traditional and drought tolerant was reported^10^. The gene (LOC_Os01g67860) appears to be a strong candidate in DD for grain yield under drought underlying QTL *qDTY*_*1.1*_. Among others, block 7.1 co-localized with QTL for cell membrane stability (DQA3/QCMS7.1, chr7:1160982–1537879). The region defined under this QTL contained a cluster of SCP-like extracellular protein family up-regulated in DD uniquely. The members of this gene family code for pathogenesis-related proteins (*OsPR1* and *OsPR1*-like genes) involved in cellular defence. Other genes under this particular region, such as LOC_Os07g03200 encoding for phytosulfokine precursors, were also uniquely up-regulated in DD, which are needed for cell differentiation and proliferation. This gene has been reported to be up-regulated in response to cell wall degrading enzyme LipA and may be involved in cellular defence^73^. Similarly, genes under QTL number 95 designated for cell membrane stability (QCMS12.1, chr12:21040696–24586392) also showed presence of an up-regulated cluster of Pathogen related Bet v I protein family amongst other uniquely up-regulated genes in DD, such as metallothionin, osmotin, MYB transcription factor. Interestingly, we found that gene coding for 9-cis-epoxycarotenoid dioxygenase (*OsNCED5*, LOC_Os12g42280) involved in ABA biosynthesis pathway was found to be differentially expressed and also to be included in QTL number 96 designated for ABA content (chr12:24398623–26677812). Thus, these predicted regions may harbour candidate genes of increased functional importance to alleviate drought stress in rice.

Finally, it can be concluded that, in all probability, it is the faster sensing mechanism and kinetics by which DD cultivar is able to activate its detoxification signalling network effectively, involving both ABA dependent and independent pathways, such as JA, auxin and ROS signalling, that allow it to hasten its adaptation to the adverse abiotic stress conditions, making it a drought tolerant cultivar. Even though there might be several genes unique to IR20 such as *OsFBK1*, which prove to be candidates for conferring drought tolerance, this response towards stress by DD appears to be an evolved response to cope up with water deficit stress, modulated by protection of cell organelles and membranes from oxidative stress and the simultaneous induction of stress responsive genes, which is evident from the large number and functional character of DEGs after 3 h of stress. A robust regulation of both ABA dependent and ABA independent pathways appears to be critical for enhancing drought stress tolerance in rice whereas reliance on just ABA inducible genes may lead to susceptible behaviour under stress. Indigenous drought tolerant rice cultivars may have evolved over time strengthening these pathways specifically leading to faster acclimation under stress.

Additionally, the data generated in this study could be used as a resource to identify genes (from both the cultivars) suitable for genetic manipulation, thereby generating or enhancing the drought responsiveness of the susceptible albeit high-yielding rice cultivars. Our study also shows that one must exercise caution and probably educated choices while selecting genes from contrasting cultivars for enhancing stress responsiveness of crop plants, as stress effective genes could be present in either parent and not necessarily in the tolerant cultivar only. Some of these genes mapped to the known QTLs could be validated and used for marker assisted breeding for conferring stress tolerance in high yielding but susceptible rice cultivars.

## Methods

### Plant material and growth conditions

Approximately 50–60 seeds of Dhagaddeshi (DD) and IR20 were surface sterilized by 0.1% HgCl_2,_ washed repeatedly with autoclaved MQ, and kept at 28 ± 1 °C for 16 h for imbibition. Seedlings were grown hydroponically on Yoshida medium for seven days in a culture room maintained at 28 ± 1 °C. On the eighth day, the seedlings were either used for physiological analyses or placed in triplicates on 3 mm Whatmann sheets for imparting desiccation stress and kept in culture room conditions at 28 ± 1 °C for 3 h and 6 h^13^. These tissues were harvested (after removing the seeds) at the specified time-points, snap frozen in liquid nitrogen and stored at −80 °C till further use. The control samples were kept in Yoshida medium under the same conditions and harvested at the specified time points along with the samples subjected to desiccation stress. The same procedure was followed for the other cultivars.

### Physiological analysis

Relative water content (RWC) was measured from control and stressed leaf tissue following the method described by^74^. Leaf tissue from 3 seedlings per sample was cut into ½ cm pieces, floated on deionized water in closed petri dishes at room temperature for 4 hours, blotted on tissue to drain excess water and weighed. The leaf pieces were dried in oven at 80 °C for 24 hours. The RWC was calculated using the formula: RWC (%) = [(FW − DW)]/[(TW − DW)] * 100, where FW is the initial fresh weight of leaf tissues, TW is the turgid weight of tissues after 4 hours of incubation in water at room temperature and DW is dry weight after oven drying at 80 °C for 24 hours. Measurements for cell membrane stability (CMS) were carried as described by ref. 4. Briefly, seedlings (3 each) were washed with deionized water and then given stress treatment. Stressed (T) and control (C) leaf tissue discs were floated on deionized water for 24 hours in dark and the conductance (T1 and C1) was measured with a conductivity meter (CYBERSCAN CON 11 Conductivity/TDS/^o^C meter, Eu Tech Instruments, Singapore). The samples were then autoclaved for 15 minutes and again the conductance was measured from cooled samples (T2 and C2). The membrane stability was calculated as CMS in % = [(1 − (T1/T2))/(1 − (C1/C2))] * 100.

### Estimation of ABA, free proline, total chlorophyll and carotenoids

The ABA content for whole seedlings was estimated using Phytodetek ABA test kit (AGDIA) by competitive ELISA method. Sample preparation for ABA estimation was done as described by ref. 75. Briefly, one-week-old rice seedlings were ground in liquid nitrogen and extracted overnight at 4 °C in 1 mL of extraction buffer containing 20 mL L^−1^ acetic acid, 90% methanol and 10 mg L^−1^ butylated hydroxytoluene. The supernatant after centrifugation was collected and evaporated to 100 μl final volume using a speed vac. Dilutions were made in 1× TBS buffer and further quantification was done as per manufacturer’s instructions. Estimation of free proline content was done colorimetrically according to ref. 76. Total chlorophyll and carotenoid content was determined spectrophotometrically using the non-maceration DMSO method as given by ref. 77. Leaf tissue (0.03 g) was incubated with 3 mL DMSO in dark overnight and then centrifuged at maximum speed for 1 minute. The supernatant was used for measuring absorbance at 665, 649 and 480 nm with UV-Vis spectrophotometer (Hitachi U-2810, Japan). Chlorophyll and carotenoid content was calculated according^77^.

### Microarray hybridization and data analysis

Isolation of total RNA was done as described previously^78^. Target preparation, hybridization to arrays, washing, staining and scanning were carried out per manufacturer’s instructions (GeneChip^®^ 3’ IVT Express Kit User Manual, 2008, Affymetrix). Affymetrix GeneChip Operating Software 1.2.1 was used for washing and scanning in Fluidics Station 450 (Affymetrix) and Scanner 3000 (Affymetrix), respectively. For data analysis, the probe intensity (.cel) files of all 18 chips were imported into ArrayAssist^®^ (Stratagene). Normalization of data was carried out by using the GC-RMA algorithm (Gene Chip Robust Multiarray Analysis) implemented in the software. The genes after ANOVA analysis showing change of at least two-fold at a p-value of <0.05 under drought conditions were identified as differentially expressed genes. Further, these genes were annotated as per TIGR version 7. Redundant probe-sets were removed and genes with unknown functions were manually annotated by sequence retrieval (NCBI, KOME and TIGR) and domain search using SMART and Pfam databases. Further information was derived from Gene Ontology (GO), InterPro, Uniprot and published data. The gene lists were then divided into functional groups based on those previously suggested^25^,^26^.

The GC-RMA normalized data have been submitted to GEO under accession number GSE41647.

### Real-time PCR analysis

To confirm the differential expression of drought-responsive genes highlighted in DD and IR20 after microarray data analysis, real-time PCR analysis for each time point of 1 h, 2 h, 3 h, 4 h, 5 h and 6 h was performed using gene-specific primers as described earlier^78^. The primer sequences are listed in Supplementary Table S2. At least two biological replicates of each sample and three technical replicates of each biological replicate were used for real-time PCR analysis (*Ubiquitin* as the control gene) in the LightCycler© 480II Real Time system (Roche, Germany). For cultivar specific real-time PCR analysis, the procedure followed was like the one carried out for DD and IR20.

### Functional validation of *OsFBK1*

The CDS of *OsFBK1* (LOC_Os01g47050) was amplified from the KOME clone AK121359 and cloned in the binary vectors: pCTB (for *Arabidopsis*) and pB4NU (for rice). For the generation of RNAi lines, a 298 bp sequence was amplified from the 3’UTR region of *OsFBK1* and cloned in the pANDA destination vector^79^ by employing the Gateway™ technology (pENTR™ Directional TOPO^®^ cloning kit, and LR clonase Enzyme mix II kit, Invitrogen Inc. USA). *Agrobacterium*-mediated transformation was carried out in *Arabidopsis* (Col-0) and Pusa Basmati 1 as per the protocols described earlier^80^,^81^ respectively. For *Arabidopsis* mutant analysis, the homozygous seeds of the *hws* mutants, SALK_058990 and SALK_088349 (ABRC) were used. For germination assays, homozygous seeds of the mutant (*hws*), knock-down (*OsFBK1*^*RNAi*^) and over-expression (*OsFBK1*^*ox*^) lines of *Arabidopsis* and rice respectively, were grown on MS medium supplemented with ABA: 1, 5 and 10 μM (*Arabidopsis*) and 1, 2 and 5 μM (rice), and germination counted on Day 3 after inoculation. Performance of the transgenics on prolonged ABA exposure was analysed on 1 μM ABA (*Arabidopsis*)/2 μM ABA (rice) for 10-days in light under culture room conditions (22 ± 1 °C for *Arabidopsis* and 28 ± 1 °C for rice). Real-time PCR of 7-day-old seedlings of IR64 was carried out after exposure to the following stresses for 3 h at 28 ± 1 °C under light: 200 mM NaCl solution, temperature stresses at 10 °C and 37 °C, 10 μM ABA solution, 20 μM JA solution, 1 μM BR solution, and dehydration stress by placing the seedlings on 3 mm Whatmann sheets.

### QTL and pathway analysis

Based on observed physiological traits for the two rice cultivars used in this study, Quantitative Trait Loci (QTLs) data were retrieved from Gramene and Tropgene databases and other published literature as listed in Supplementary Table S3. The physical positions of delimiting molecular markers associated with the QTLs were retrieved using available *indica* and *japonica* rice reference genome sequence^82^,^83^,^84^. Comparison of physical coordinates of markers on rice chromosomes and gene loci (TIGR Genome Browser version 7) highlighted in microarray expression data allowed mining of differentially expressed gene loci underlying the QTLs. The mapping of DEGs on rice chromosome was performed using MicroArray Data Interface for Biological Annotation (MADIBA)^85^ (http://madiba.bi.up.ac.za/).

To generate the metabolic profile of the differentially expressed genes, the microarray expression data were integrated with metabolic pathway data available at Gramene RiceCyc database (version 3.2)^86^. Pathway tools version 16.5 was used to identify significantly enriched genes for pathways (P-value ≤ 0.05) and further pathways were reconstructed using Microsoft PowerPoint. The drought signalling network pathway was reconstructed in the Adobe Illustrator CS5 Web premium software.

## Additional Information

**Accession codes:** GSE41647.

**How to cite this article:** Borah, P. *et al*. Analysis of drought-responsive signalling network in two contrasting rice cultivars using transcriptome-based approach. *Sci. Rep.* **7**, 42131; doi: 10.1038/srep42131 (2017).

**Publisher's note:** Springer Nature remains neutral with regard to jurisdictional claims in published maps and institutional affiliations.

## Supplementary Material

Supplementary Information

Supplementary Data 1

Supplementary Data 2

## Figures and Tables

**Table 1:** Expression levels of key genes involved in drought stress responses.

Gene	MSU Locus ID	Fold change [3hDD] vs [CtrlDD]	Fold change [3hIR20] vs [CtrlIR20]
*OsDREB1A*	LOC_Os09g35030	7.51	2.36
*OsDREB1B*	LOC_Os09g35010	7.56	9.74
*OsDREB1F*	LOC_Os01g73770	—	5.85
*OsDREB2A*	LOC_Os01g07120	14.94	3.89
*OsDREB2B*	LOC_Os05g27930	12.81	2.97
*OsLEA1a*	LOC_Os01g06630	21.26	4.87
*OsLEA3-2*	LOC_Os03g20680	324.57	288.03
*OsLEA3-1*	LOC_Os05g46480	779.76	21.13
*OsLEA4*	LOC_Os06g02040	258.72	26.50
*OsNAC19/SNAC1/OsNAC9*	LOC_Os03g60080	52.71	19.11
*OsNAC2/OsTIL1*	LOC_Os04g38720	3.41	—
*OsNAC4*	LOC_Os01g60020	13.64	6.50
*OsNAC5*	LOC_Os11g08210	8.53	7.32
*OsNAC6/SNAC2*	LOC_Os01g66120	13.33	5.75
*OsNAC10*	LOC_Os11g03300	183.01	31.62
*OsVP1*	LOC_Os01g68370	9.89	—
*OREB1/OsABI5/OsABF1/OsbZIP10*	LOC_Os01g64000	3.18	—
*OsbZIP12*	LOC_Os01g64730	25.66	7.25
*no apical meristem (NAM) protein*	LOC_Os01g66120	13.33	5.75
*OsRDCP1*	LOC_Os04g44820	7.76	2.85
*OsOAT*	LOC_Os03g44150	3.01	—
*DRO1*	LOC_Os09g26840	3.73	—
*OSISAP1*	LOC_Os09g31200	6.64	—
*OsNCED1*	LOC_Os02g47510	−4.26	—
*OsNCED4*	LOC_Os07g05940	601.40	309.87
*OsNCED5*	LOC_Os12g42280	41.99	138.42
*ABA8ox1*	LOC_Os02g47470	10.18	3.0273
