# Supplementary material for: Analysis of drought-responsive signalling network in two contrasting rice cultivars using transcriptome-based approach
Source: Sci Rep. 2017 Feb 9;7:42131. doi: 10.1038/srep42131 (PMC5299611; doi:10.1038/srep42131)
Supplement: Supplementary Information [file srep42131-s1.pdf]

Supplementary Information

Analysis of drought-responsive signalling network in two contrasting rice cultivars using transcriptome-based approach

Pratikshya Borah, Eshan Sharma , Amarjot Kaur , Girish Chandel , Trilochan Mohapatra , Sanjay Kapoor and Jitendra P. Khurana

Supplementary Information

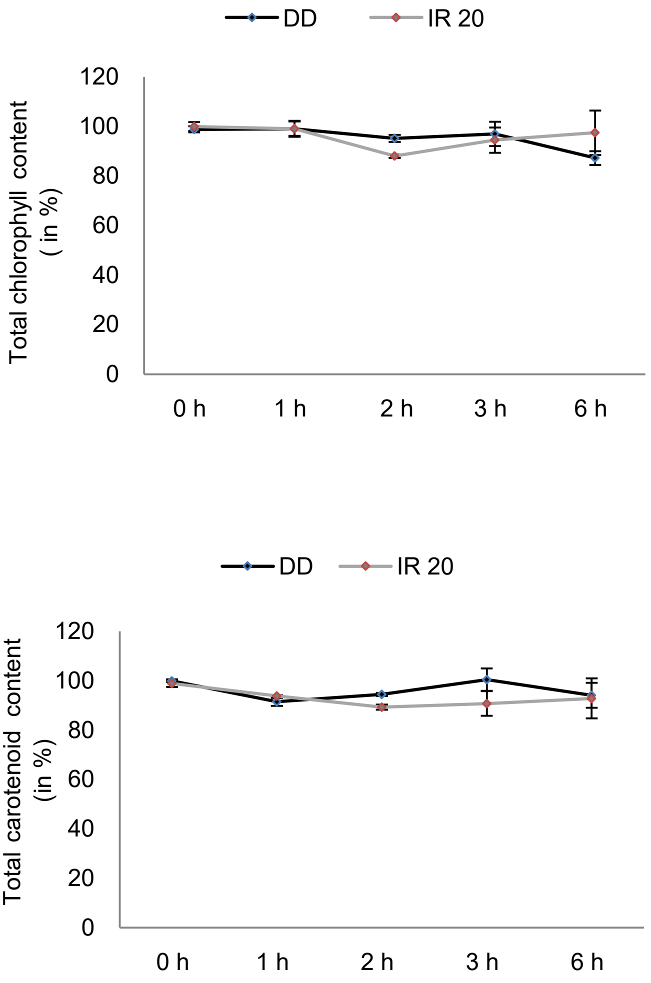

Supplementary fig. S1: Comparative analysis of total chlorophyll and carotenoid content in Dhagaddeshi and IR20. All the experiments were done in triplicates and the mean values (± SE) were plotted against duration of drought stress treatment in hours .

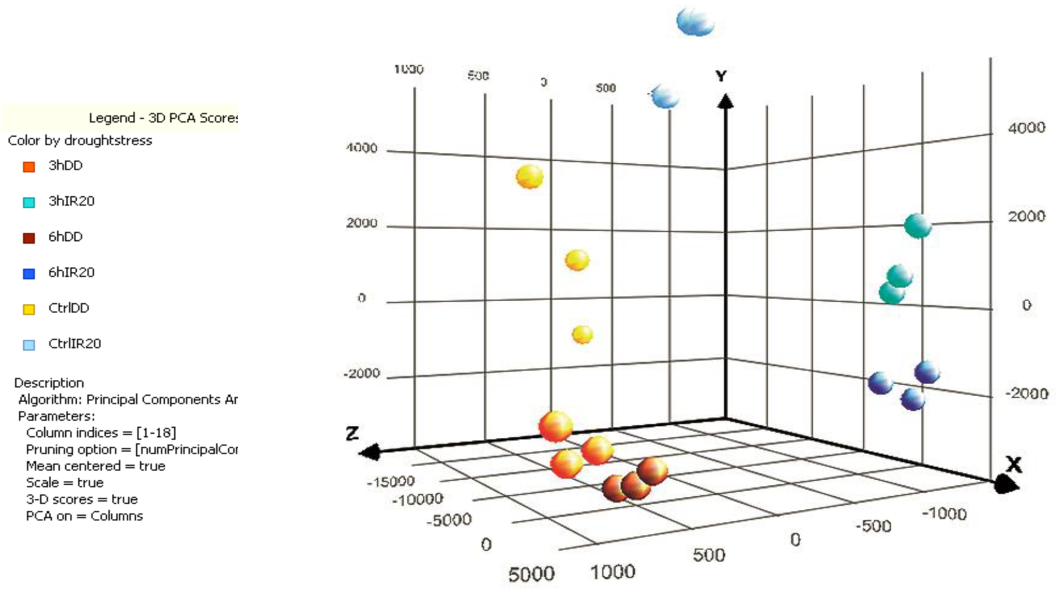

Supplementary fig. S2: Principal Component Analysis (PCA) of the eighteen chips used for the present study. Distinct clusters were formed by the replicates of the two varieties. Colour codes for different samples are shown in square boxes.

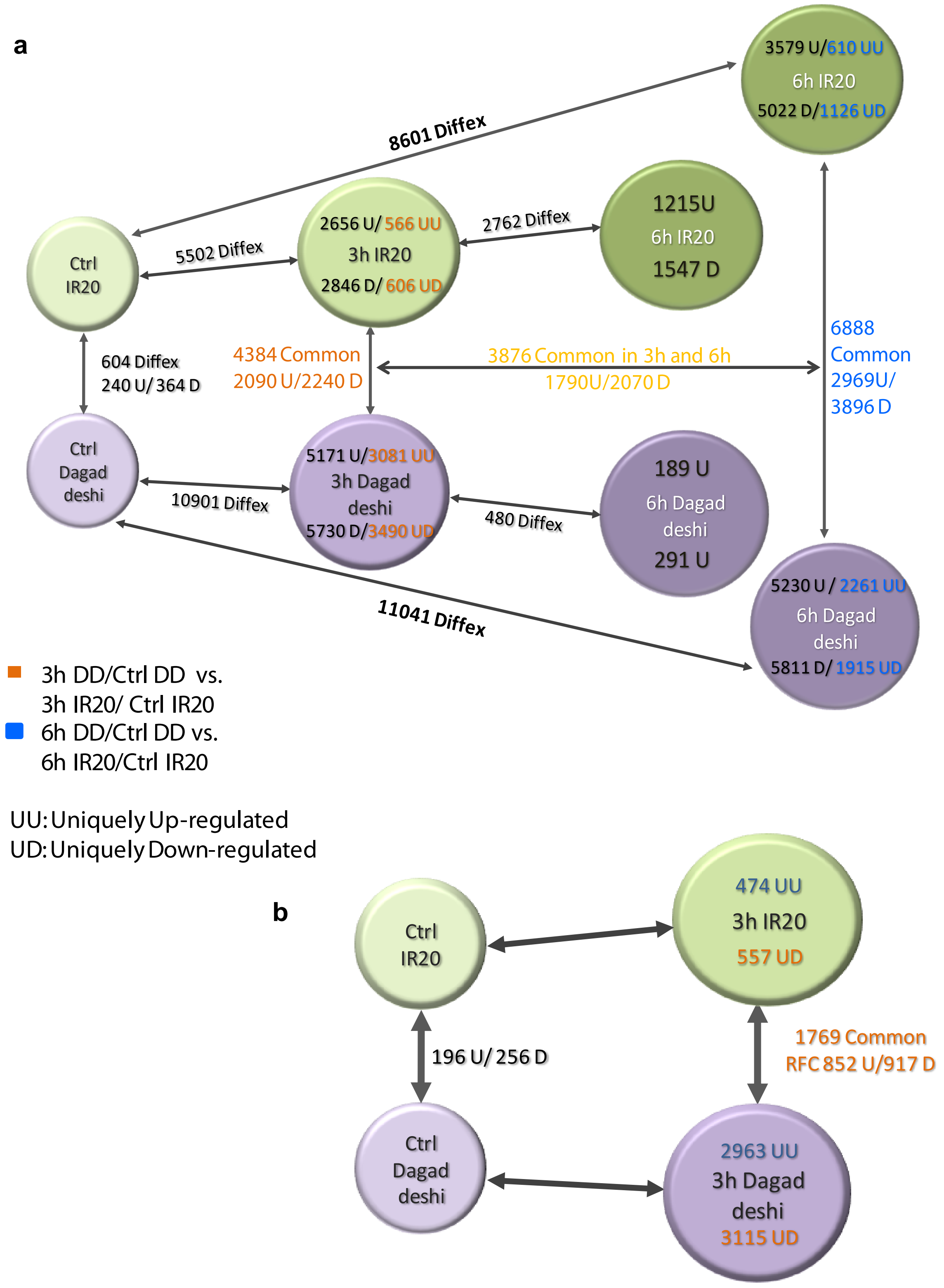

Supplementary fig. S3 a: Diffex analysis of the various samples analyzed. The number of probe-sets detected in each group has been shown. Uniquely up-regulated/down-regulated probe-sets have been colour-coded according to the 3 h and 6 h groups as given in the legend along with the figure. **3 b:** Number of genes highlighted in IR20 and DD at 3 h stress after manual curation. RFC – relative fold change.

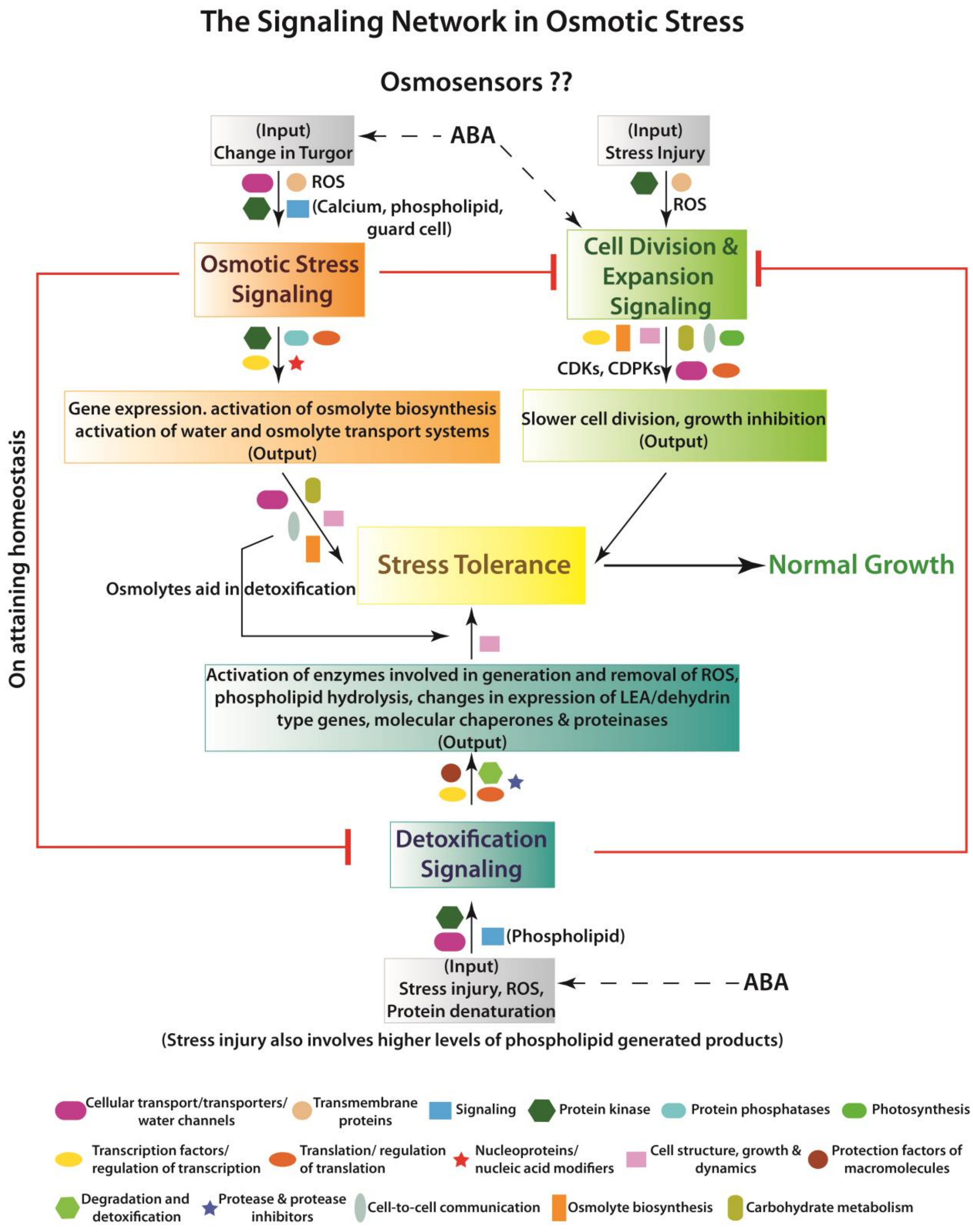

Supplementary fig. S4: The drought signalling network as predicted to operate in plants. The categories of the input and output signal players have been defined to accommodate the genes/proteins with similar functions. These broad categories have been denoted by the symbols shown in the figure and their positions in the network have been deduced from clues given in past literature. Each pathway has its own inputs and outputs, although the key players in the output signalling overlap with each other in terms of function. The nature of osmosensors is yet unknown.

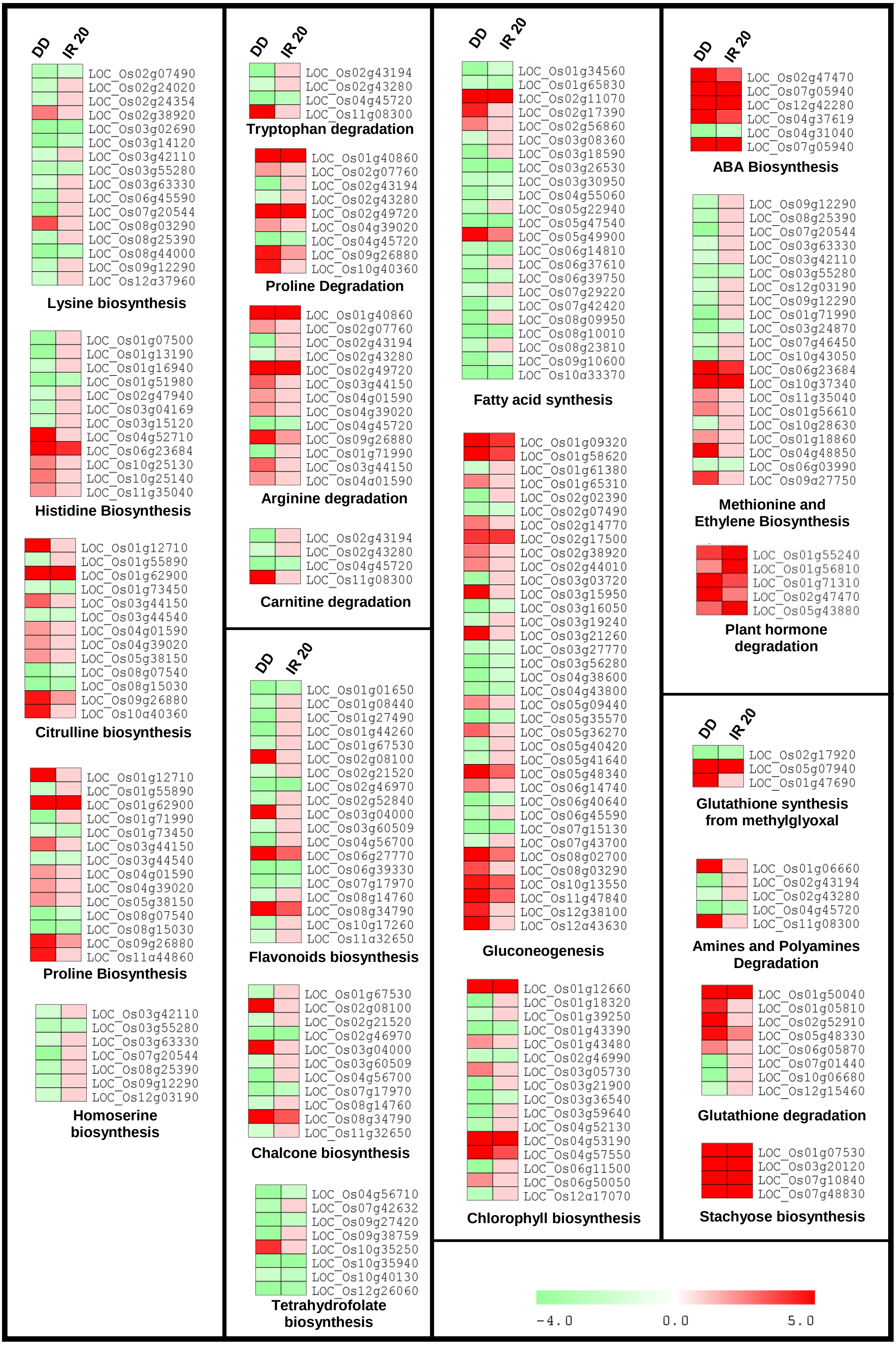

Supplementary fig. S5: The metabolic pathways enriched in differentially expressed genes of Dagad deshi under 3 h drought stress are shown with heatmaps representing their expression profile.

Supplementary Table S1: Details of genes selected for Real-time PCR analysis

LOCUS ID	CATEGORY	DESCRIPTION	Microarray fold change [3hDD/CtrlDD] vs [3hIR20/CtrlIR20]	
LOC_Os01g14440	Transcription factors/regulation of transcription	OsWRKY1v2	2.45640	Up
LOC_Os01g68370	Transcription factors/regulation of transcription	B3 DNA binding domain containing protein	2.64664	Up
LOC_Os02g32520	Stress induced	ERD1 protein, chloroplast precursor	6.66779	Up
LOC_Os03g59440	Cell structure, growth and dynamics	dirigent	-5.58523	Down
LOC_Os06g45840	Transcription factors/regulation of transcription	MYB transcription factor	2.77013	Up
LOC_Os08g43090	Transcription factors/regulation of transcription	bZIP family transcription factor	3.90588	Up
LOC_Os01g43774	Degradation and detoxification	cytochrome P450 72A1	32.58873	Up
LOC_Os12g41600	Signalling	OsSAUR57 - Auxin-responsive SAUR gene family member	-2.53978	Down
LOC_Os12g43140	Protection factors of macromolecules	late embryogenesis abundant protein D-34	7.04348	Up
LOC_Os01g72130	Degradation and detoxification	glutathione S-transferase	36.65365	Up
LOC_Os04g33150	Stress induced	desiccation-related protein PCC13-62 precursor	15.21382	Up
LOC_Os09g35030	Stress induced	DREB	3.17927	Up
LOC_Os06g04090	Transcription factors/regulation of transcription	no apical meristem protein, putative, expressed	-3.54494	Down
LOC_Os01g64730	Transcription factors/regulation of transcription	bZIP transcription factor domain containing protein, expressed	3.53626	Up

Supplementary Table S2: List of primers

**Primer**	**Sequence**
RT-FP-LOC_Os01g14440	TCCGAAGCGCCAATCATC
RT-RP-LOC_Os01g14440	GCCATCTTCTGACCGTACTTCCT
RT-FP-LOC_Os01g68370	CGACGTTGGCATGATGATAGA
RT-RP-LOC_Os01g68370	GGTACAGTCGTCCGCCTGAT
RT-FP-LOC_Os02g32520	CAGCTGCAAGCGGGTGTT
RT-RP-LOC_Os02g32520	ATGAAGCTGCAGCCCATGTT
RT-FP-LOC_Os03g59440	GCTCACTGATGGCCCCTACA
RT-RP-LOC_Os03g59440	CGGGTTCCGCGAGTACAC
RT-FP-LOC_Os06g45840	CCTCCACCCAGGCCAAA
RT-RP-LOC_Os06g45840	TTAGAGGCCTTCTGTGGATATGG
RT-FP-LOC_Os08g43090	TGCCGAGATCGCTTTGAC
RT-RP-LOC_Os08g43090	AGACTGCCGATTTGCCAA
RT-FP-LOC_Os01g43774	GAAGTTCGTCATTCCGGGTTAC
RT-RP-LOC_Os01g43774	TCCTCCGCATCCTTCTGTTC
RT-FP-LOC_Os12g41600	GCCGATCGCCGTCAACT
RT-RP-LOC_Os12g41600	TTGTCCAGCATCCACAGTAGGT
RT-FP-LOC_Os12g43140	TGATGAAGGACGTGGTGGG
RT-RP-LOC_Os12g43140	AAACCATCCTCCCCTCGTTC
RT-FP-LOC_Os01g72130	GATCCCGTTCGTGCAAATG
RT-RP-LOC_Os01g72130	CCTTTGCTTCCCTCACGAAAC
RT-FP-LOC_Os04g33150	TGGAGACGGCGATGAACA
RT-RP-LOC_Os04g33150	AGGAAGTTGAGGCTGTTCTCGTA
RT-FP-LOC_Os09g35030	TGGCGTTCGAACTGGA
RT-RP-LOC_Os09g35030	GCGTAGTACAGGTCCCA
RT-FP-LOC_Os06g04090	GTGCAGGATTGGGTCAGG
RT-RP-LOC_Os06g04090	CCCGTCGGGTACTTCTTGT
RT-FP-LOC_Os01g64730	TCGCTGGTCACGCAACTC
RT-RP-LOC_Os01g64730	TGATGTTGCTCCTCCTGTTCTTT
OsFBK1 pB4NU	F: 5’ ATAGGATCCATGGAGGAGCAGA 3’<br>R: 5’ CAGGGTACCTTAAGTAATATCAA 3’
OsFBK1 pCTB	F: 5’ ATATCTAGAATGGAGGAGCAGAAGCT 3’<br>R: 5’ CAGGAGCTCTTAAGTGGTAATATCAA 3’
OsFBK1-RT	F: 5’ CACCTGTGGACGTCTGCTTAAC 3’<br>R: 5’ TCAGGTCTGTTCTGTTTTGCAATC 3’
Ubiquitin-RT	F: 5’ ACCACTTCGACCGCCACTACT 3’<br>R: 5’ ACGCCTAAGCCTGCTGGTT 3’
OsFBK1-RNAi	F: 5’ CACCAGATTGTTGCGTCACAT 3’<br>R: 5’ ATATGGACAGGTTCTAAGGCC 3’

Supplementary Table S3: List of QTLs analysed in the present study

QTL NO.	QTL ID	Character/ trait	Chromosome no.	Start position (in bp)	End position (in bp)	Source
1	DQA1	Cell membrane stability	1	37887264	37887562	GRAMENE DATABASE
2	AQAN003	Drought tolerance	1	32060549	32061040	GRAMENE DATABASE
3	AQHP057	Drought tolerance	1	29184260	29184844	GRAMENE DATABASE
4	CQH1	Osmotic adjustment capacity	1	5094276	5095699	GRAMENE DATABASE
5	CQAV1	Osmotic adjustment capacity	1	34937981	41541798	GRAMENE DATABASE
6	AQDX001	Osmotic adjustment capacity	1	4424392	4424515	GRAMENE DATABASE
7	AQDX002	Osmotic adjustment capacity	1	5094276	7445919	GRAMENE DATABASE
8	AQDX003	Osmotic adjustment capacity	1	35196573	36734272	GRAMENE DATABASE
9	QCMS1.1	Cell membrane stability	1	31367453	37887562	TROPGENE DATABASE
10	-NA-	Drought resistance	1	32987224	36470382	Salunkhe et. al., 2011
11	qRGV-I	Relative germination vigor	1	26814428	32987357	You Jun et. al., 2006
12	qLRS- 1	Leaf rolling scores	1	26814428	32987357	You Jun et. al., 2006
13	qDTY1.1	Grain yield	1	38895261	40580568	Ghimire et. al. , 2012
14	qDTH1.2	Plant height	1	36731793	38892963	Ghimire et. al. , 2012
15	AQHP058	Drought tolerance	2	10503368	10503846	GRAMENE DATABASE
16	AQHP066	Drought tolerance	2	29761981	29762453	GRAMENE DATABASE
17	AQHP067	Drought tolerance	2	27034342	27035328	GRAMENE DATABASE
18	AQHP068	Drought tolerance	2	10503368	19866086	GRAMENE DATABASE
19	CQH6	Osmotic adjustment capacity	2	25865334	28354640	GRAMENE DATABASE
20	AQDX004	Osmotic adjustment capacity	2	7706705	17485139	GRAMENE DATABASE
21	1391	ABA content	2	30270605	30270605	TROPGENE DATABASE
22	qLRS-2	Leaf rolling scores	2	7433419	11389936	You Jun et. al., 2006
23	-NA-	Seedling stage drought tolerance	2	9162844	11389936	Xu et. al., 2011
24	DQA2	Cell membrane stability	3	23088332	23088721	GRAMENE DATABASE
25	AQHP069	Drought tolerance	3	22798284	35828040	GRAMENE DATABASE
26	AQHP079	Drought tolerance	3	15469002	19412007	GRAMENE DATABASE

27	CQH12	Osmotic adjustment capacity	3	8409404	8410886	GRAMENE DATABASE
28	AQFT003	Osmotic adjustment capacity	3	9939965	12147908	GRAMENE DATABASE
29	CQAV2	Osmotic adjustment capacity	3	19411563	22799337	GRAMENE DATABASE
30	AQDX005	Osmotic adjustment capacity	3	6041028	6042440	GRAMENE DATABASE
31	QCMS 3.1	Cell membrane stability	3	21237371	23088721	TROPGENE DATABASE
32	1392	ABA content	3	26728825	26728825	TROPGENE DATABASE
33	qLRS-3	Leaf rolling scores	3	9828577	12407510	You Jun et. al., 2006
34	-NA-	Seedling stage drought tolerance	3	1240738	17713829	Xu et. al., 2011
35	AQA045	Drought susceptibility index	4	13634515	13635012	GRAMENE DATABASE
36	AQAN004	Drought tolerance	4	8610617	8611256	GRAMENE DATABASE
37	AQHP059	Drought tolerance	4	31662839	31663326	GRAMENE DATABASE
38	AQDX006	Osmotic adjustment capacity	4	24690120	24690798	GRAMENE DATABASE
39	1393	ABA content	4	31279646	31279646	TROPGENE DATABASE
40	1394	ABA content	4	20057078	22349768	TROPGENE DATABASE
41	lABA4.1_E4	Leaf ABA concentration	4	13141576	18580250	Dominique this et. al., 2010
42	AQAN001	Drought tolerance	5	27342022	28610866	GRAMENE DATABASE
43	CQAV8	Osmotic adjustment capacity	5	6132767	18875558	GRAMENE DATABASE
44	AQDX007	Osmotic adjustment capacity	5	189782	494181	GRAMENE DATABASE
45	AQDX008	Osmotic adjustment capacity	5	24316513	28610866	GRAMENE DATABASE
46	qRGR-5	Relative germination rate	5	23976333	27313476	You Jun et. al., 2006
47	-NA-	Seedling stage drought tolerance	5	22680950	23955706	Xu et. al., 2011
48	AQHP061	Drought tolerance	6	2560318	2561213	GRAMENE DATABASE
49	AQHP082	Drought tolerance	6	6718648	9537772	GRAMENE DATABASE
50	1395	ABA content	6	29503726	29503726	TROPGENE DATABASE
51	-NA-	Seedling stage drought tolerance	6	25825428	28599181	Xu et. al., 2011
52	DQA3	Cell membrane stability	7	1160982	1537879	GRAMENE DATABASE
53	AQHP073	Drought tolerance	7	13074864	13075056	GRAMENE DATABASE
54	CQAV3	Osmotic adjustment capacity	7	13074864	29467498	GRAMENE DATABASE
55	CQAV4	Osmotic adjustment capacity	7	1536133	2317976	GRAMENE DATABASE
56	AQDX009	Osmotic adjustment capacity	7	19256914	19257039	GRAMENE DATABASE

57	AQDX010	Osmotic adjustment capacity	7	28209280	28209648	GRAMENE DATABASE
58	QCMS7.1	Cell membrane stability	7	1160982	1537879	TROPGENE DATABASE
59	1396	ABA content	7	26704922	26704922	TROPGENE DATABASE
60	DQA4	Cell membrane stability	8	18994607	19052586	GRAMENE DATABASE
61	DQA5	Cell membrane stability	8	27734995	27735542	GRAMENE DATABASE
62	AQAN005	Drought tolerance	8	20094533	20094695	GRAMENE DATABASE
63	CQH28	Osmotic adjustment capacity	8	18994607	19052586	GRAMENE DATABASE
64	AQFT004	Osmotic adjustment capacity	8	19051713	20662645	GRAMENE DATABASE
65	CQAV5	Osmotic adjustment capacity	8	4105519	4106001	GRAMENE DATABASE
66	CQAV6	Osmotic adjustment capacity	8	17437513	25592993	GRAMENE DATABASE
67	AQDX011	Osmotic adjustment capacity	8	682963	683186	GRAMENE DATABASE
68	AQDX012	Osmotic adjustment capacity	8	21142348	22472031	GRAMENE DATABASE
69	QCMS8.1	Cell membrane stability	8	18995081	19052586	TROPGENE DATABASE
70	QCMS8.2	Cell membrane stability	8	27735050	27895639	TROPGENE DATABASE
71	qRGV-8	Relative germination vigor	8	26492216	28075494	You Jun et. al., 2006
72	DQA6	Cell membrane stability	9	7222547	9216863	GRAMENE DATABASE
73	DQA7	Cell membrane stability	9	18810067	18810331	GRAMENE DATABASE
74	AQHP063	Drought tolerance	9	20481606	20482133	GRAMENE DATABASE
75	CQH30	Osmotic adjustment capacity	9	9216346	10906419	GRAMENE DATABASE
76	-NA-	ABA accumulation	9	19559036	19562133	GRAMENE DATABASE
77	QCMS9.1	Cell membrane stability	9	7222547	7887602	TROPGENE DATABASE
78	QCMS9.2	Cell membrane stability	9	12643255	18810331	TROPGENE DATABASE
79	1397	ABA content	9	14362062	14362062	TROPGENE DATABASE
80	1398	ABA content	9	18905061	18905061	TROPGENE DATABASE
81	qLRS-9	Leaf rolling scores	9	10784216	12348411	You Jun et. al., 2006
82	DRO1	Deep rooting 1	9	15972483	16580764	Uga et. al., 2011
83	AQHP076	Drought tolerance	10	15843719	15844682	GRAMENE DATABASE
84	AQHP086	Drought tolerance	10	12900279	12903065	GRAMENE DATABASE
85	AQDX014	Osmotic adjustment capacity	10	21402080	21402437	GRAMENE DATABASE
86	qRGV-10	Relative germination vigor	10	18014265	21066729	You Jun et. al., 2006

87	DQA8	Cell membrane stability	11	19565059	19565672	GRAMENE DATABASE
88	AQHP064	Drought tolerance	11	19565059	19565672	GRAMENE DATABASE
89	AQHP078	Drought tolerance	11	4413928	4415836	GRAMENE DATABASE
90	QCMS11.1	Cell membrane stability	11	19565059	22014851	TROPGENE DATABASE
91	qLRC-11	Leaf rolling scores	11	18407879	19173094	You Jun et. al., 2006
92	DQA9	Cell membrane stability	12	18867450	21199237	GRAMENE DATABASE
93	AQA046	Drought susceptibility index	12	26017140	27489485	GRAMENE DATABASE
94	AQAN002	Drought tolerance	12	19628443	19628925	GRAMENE DATABASE
95	QCMS12.1	Cell membrane stability	12	21040696	24586392	TROPGENE DATABASE
96	lABA12.1_E3	Leaf ABA concentration	12	24398623	26677812	Dominique this et. al., 2010
97	qtl12.1	Grain yield under drought stress	12	14106460	17395485	Bernier et. al., 2007

*-NA- indicates QTL ID not assigned in related published literature.

Supplementary qDTY1.1 genes list

List of genes **up-regulated** under *qDTY1.1*		
Gene ID	Putative_Function	Fold Change
LOC_Os01g66120	No apical meristem (NAM) protein, putative, expressed	2.316891813
LOC_Os01g66250	S-locus-like receptor protein kinase, putative, expressed	5.0359077
LOC_Os01g66280	transcriptional regulator, putative, expressed	2.2694902
AK120651	higher plant specific, expressed protein	8.071829
AK107053	rice specific, expressed protein	5.2497377
LOC_Os01g67040	OsRhmbd5 - Putative Rhomboid homologue, expressed	2.4940972
LOC_Os01g67220	Os1bglu4 - beta-glucosidase-like protein without signal sequence, expressed	2.16772
LOC_Os01g67250	Rad21 / Rec8 like protein, putative, expressed	2.2528064
LOC_Os01g67330	nucleotide-sugar transporter family protein, putative, expressed	2.4367204
LOC_Os01g67364	retrotransposon protein, putative, Ty3-gypsy subclass, expressed	3.5220156
LOC_Os01g67860	Fructose-bisphosphate aldolase, cytoplasmic isozyme, putative, expressed	120.40781
LOC_Os01g68290	monocot specific, expressed protein	13.836969
LOC_Os01g68300	monocot specific, high expression in seed developmental stages, expressed protein	5.3276167
LOC_Os01g68330	antigen peptide transporter-like 1, chloroplast precursor, putative, expressed	6.8262415
LOC_Os01g68330	antigen peptide transporter-like 1, chloroplast precursor, putative, expressed	2.5959706
LOC_Os01g68370	B3 DNA binding domain containing protein, expressed	9.892861
LOC_Os01g68660	cysteine proteinase inhibitor precursor protein, putative, expressed	2.7097948
LOC_Os01g68720	keratin, type I cytoskeletal 9, putative, expressed	17.617886
LOC_Os01g68730	RNA-binding protein FUS, putative, expressed	5.879446
LOC_Os01g68740	keratin, type I cytoskeletal 9, putative, expressed	3.9951746
LOC_Os01g68750	Adaptin N terminal region family protein, expressed	4.7159333
LOC_Os01g68890	P21-Rho-binding domain containing protein, putative, expressed	5.254818
LOC_Os01g68960	conserved hypothetical protein	12.528228
LOC_Os01g69040	zinc finger, C3HC4 type domain containing protein, expressed	2.7662055
LOC_Os01g69100	Pfam-B_10205, higher plant specific, expressed protein	4.9594684
LOC_Os01g69110	expressed protein	2.238363
LOC_Os01g69910	calmodulin-binding transcription activator, putative, expressed	2.031057
LOC_Os01g70090	enoyl-CoA hydratase/isomerase family protein, putative, expressed	3.5361614

List of genes **down-regulated** under *qDTY1.1*		
Gene ID	Putative_Function	Fold Change
LOC_Os01g66860	serine/threonine protein kinase, putative, expressed	4.7259517
LOC_Os01g66980	Trigger_N (Bacterial trigger factor protein (TF)), expressed protein	10.208614
LOC_Os01g67010	DOMON domain, Eukaryotic cytochrome b561, transmembrane domain, expressed protein	7.1075363
LOC_Os01g67030	auxin-responsive protein, putative, expressed	2.5616295
LOC_Os01g67090	IQ calmodulin-binding motif domain containing protein, expressed	2.9184923
LOC_Os01g67190	ribonuclease T2 family domain containing protein, expressed	2.3764482
LOC_Os01g67300	monocot specific, expressed protein	5.8456087
LOC_Os01g67390	POEI32 - Pollen Ole e I allergen and extensin family protein precursor, expressed	5.434323
LOC_Os01g67520	VTC2, putative, expressed	3.186001
LOC_Os01g67530	AMP-binding enzyme, putative, expressed	2.6985254
LOC_Os01g67550	FAD binding domain containing protein, expressed	3.3708196
LOC_Os01g67590	oligopeptidase, putative, expressed	2.4717379
LOC_Os01g67720	ABC1 family domain containing protein, putative, expressed	2.0123026
LOC_Os01g67740	chromosome segregation protein, putative, expressed	2.597981

LOC_Os01g67750	expressed protein	2.4500244
LOC_Os01g67870	expressed protein	2.276179
LOC_Os01g67880	vacuolar protein sorting-associated protein 53, putative, expressed	2.3624206
LOC_Os01g68104	ZOS1-21 - C2H2 zinc finger protein, expressed	2.9621596
LOC_Os01g68120	DCL3, putative, expressed	2.327853
LOC_Os01g68310	sas10/Utp3 family protein	2.220906
LOC_Os01g68324	dolichyl-diphosphooligosaccharide--protein glycosyltransferase 63 kDasubunit precursor, putative, expressed	2.7443488
LOC_Os01g68450	expressed protein	2.7763205
LOC_Os01g68524	pentatricopeptide, putative, expressed	4.0752416
LOC_Os01g68580	LTPL38 - Protease inhibitor/seed storage/LTP family protein precursor, expressed	3.7003248
LOC_Os01g68589	LTPL39 - Protease inhibitor/seed storage/LTP family protein precursor, expressed	2.6549203
LOC_Os01g68598	expressed protein	6.1131263
LOC_Os01g68620	signal peptide peptidase-like 2B, putative, expressed	7.6595516
LOC_Os01g68830	plant specific, expressed protein	3.7594993
LOC_Os01g68860	zinc finger C-x8-C-x5-C-x3-H type family protein, expressed	2.3449776
LOC_Os01g68870	leucine-rich repeat receptor protein kinase EXS precursor, putative, expressed	3.078602
LOC_Os01g69020	retrotransposon protein, putative, unclassified, expressed	2.4383483
LOC_Os01g69060	hydrolase, alpha/beta fold family domain containing protein, expressed	4.596872079
LOC_Os01g69070	auxin efflux carrier component, putative, expressed	4.202376
LOC_Os01g69120	uncharacterized RNA methyltransferase pc1998, putative, expressed	6.855957
LOC_Os01g69279	expressed protein	2.3732142
LOC_Os01g69840	highly expressed in leaf, expressed protein	3.367145145
LOC_Os01g69950	50S ribosomal protein L27, chloroplast precursor, putative, expressed	4.6128807
LOC_Os01g70020	DEK C terminal domain containing protein, expressed	2.3663325
LOC_Os01g70100	zinc finger DHHC domain-containing protein, putative, expressed	2.5171962
